# Causal relationships between gut microbiota and programmed cell death protein 1/programmed cell death-ligand 1: A bidirectional Mendelian randomization study

**DOI:** 10.3389/fimmu.2023.1136169

**Published:** 2023-03-09

**Authors:** Yu-Feng Huang, Wei-Ming Zhang, Zhi-Song Wei, Huan Huang, Qi-Yan Mo, Dan-Li Shi, Lu Han, Yu-Yuan Han, Si-Kai Nong, Guo-Xiang Lin

**Affiliations:** ^1^The First Clinical College, Shanxi Medical University, Jinzhong, China; ^2^Department of Oncology, Wuming Hospital of Guangxi Medical University, Nanning, China

**Keywords:** PD-1/PD-L1, gut microbiota, bidirectional Mendelian randomization, pQTL, causality

## Abstract

**Background:**

Multiple clinical studies have indicated that the gut microbiota influences the effects of immune checkpoint blockade (ICB) therapy comprising PD-1/PD-L1 inhibitors, but the causal relationship is unclear. Because of numerous confounders, many microbes related to PD-1/PD-L1 have not been identified. This study aimed to determine the causal relationship between the microbiota and PD-1/PD-L1 and identify possible biomarkers for ICB therapy.

**Method:**

We used bidirectional two-sample Mendelian randomization with two different thresholds to explore the potential causal relationship between the microbiota and PD-1/PD-L1 and species-level microbiota GWAS to verify the result.

**Result:**

In the primary forward analysis, genus_Holdemanella showed a negative correlation with PD-1 [βIVW = -0.25; 95% CI (-0.43 to -0.07); P_FDR_ = 0.028] and genus_Prevotella9 showed a positive correlation with PD-1 [βIVW = 0.2; 95% CI (0.1 to 0.4); P_FDR_ = 0.027]; order_Rhodospirillales [βIVW = 0.2; 95% CI (0.1 to 0.4); P_FDR_ = 0.044], family_Rhodospirillaceae [βIVW = 0.2; 95% CI (0 to 0.4); P_FDR_ = 0.032], genus_Ruminococcaceae_UCG005 [βIVW = 0.29; 95% CI (0.08 to 0.5); P_FDR_ = 0.028], genus_Ruminococcus_gnavus_group [βIVW = 0.22; 95% CI (0.05 to 0.4); P_FDR_ = 0.029], and genus_Coprococcus_2 [βIVW = 0.4; 95% CI (0.1 to 0.6); P_FDR_ = 0.018] were positively correlated with PD-L1; and phylum_Firmicutes [βIVW = -0.3; 95% CI (-0.4 to -0.1); P_FDR_ = 0.031], family_ClostridialesvadinBB60group [βIVW = -0.31; 95% CI (-0.5 to -0.11), P_FDR_ = 0.008], family_Ruminococcaceae [βIVW = -0.33; 95% CI (-0.58 to -0.07); P_FDR_ = 0.049], and genus_Ruminococcaceae_UCG014 [βIVW = -0.35; 95% CI (-0.57 to -0.13); P_FDR_ = 0.006] were negatively correlated with PD-L1. The one significant species in further analysis was species_Parabacteroides_unclassified [βIVW = 0.2; 95% CI (0-0.4); P_FDR_ = 0.029]. Heterogeneity (P > 0.05) and pleiotropy (P > 0.05) analyses confirmed the robustness of the MR results.

## Introduction

Immune checkpoint blockade (ICB) therapy has been a significant breakthrough in cancer research discovery in recent years; it offers a highly effective method for enhancing anticancer effects against aggressive cancers (1). Programmed cell death protein 1 (PD-1)/programmed cell death ligand 1 (PD-L1) is one of the most high-profile target protein pairs in ICB. PD-1/PD-L1 can inhibit the proliferation and differentiation of effector T lymphocytes and prevent the presentation of neoantigens. PD-1 is mainly expressed by activated T cells, dendritic cells (DCs), B cells, and natural killer cells (NKs). However, many tumors have been shown to elevate PD-1 expression, which further helps the tumor escape from the immune system (2). Numerous cell types express PD-L1, but its expression is significantly elevated in most malignancies, which are the primary source of PD-L1 in the blood (3). PD-1/PD-L1 inhibitors can relieve the limitation of PD-1/PD-L1 and restore exhausted T cells to resume the antitumor immune reaction (4). In clinical practice, however, ICB has limited efficacy in many patients; thus, there is an urgent need to find an auxiliary treatment to promote the effect of ICB.

In recent years, numerous investigations have shown that the gut microbiota influences the efficacy of ICB (5, 6). The gut microbiota consists of approximately 4 × 10^13^ symbiotic bacteria, protozoa, fungi, archaea, and viruses. It can affect numerous physiological systems, such as metabolism, inflammatory processes, and immune responses (7, 8). Previous research links the microbiota to the toxicity and efficacy of cancer treatments and the processes of carcinogenesis with specific taxa of bacteria (9). Identifying microbial taxa that directly or indirectly produce anticancer activities is essential for developing a microbiome-based combinatory therapy that can enhance the overall rate of response to anti–PD-1/PD-L1 therapy. A few bacterial genera/species are enriched in patients with favorable clinical outcomes (6, 10). However, due to the influence of reverse causality and confounders, the causal association between PD-1/PD-L1 and the microbiota has not been verified and many potentially related microbes associated with PD-1/PD-L1 therapy have not been explored (8). Therefore, it is essential to initiate a relevant genetic-level study.

Mendelian randomization (MR) is a genetic epidemiology method that utilizes human genetic variation known to influence modifiable exposures as instrumental variables (IVs) to infer the causal effect of an exposure on an outcome; it can eliminate confounding bias and is advantageous for separating the causal pathways of phenotypically grouped risk variables that are hard to randomize or susceptible to measurement error (11).

**Figure 1:**
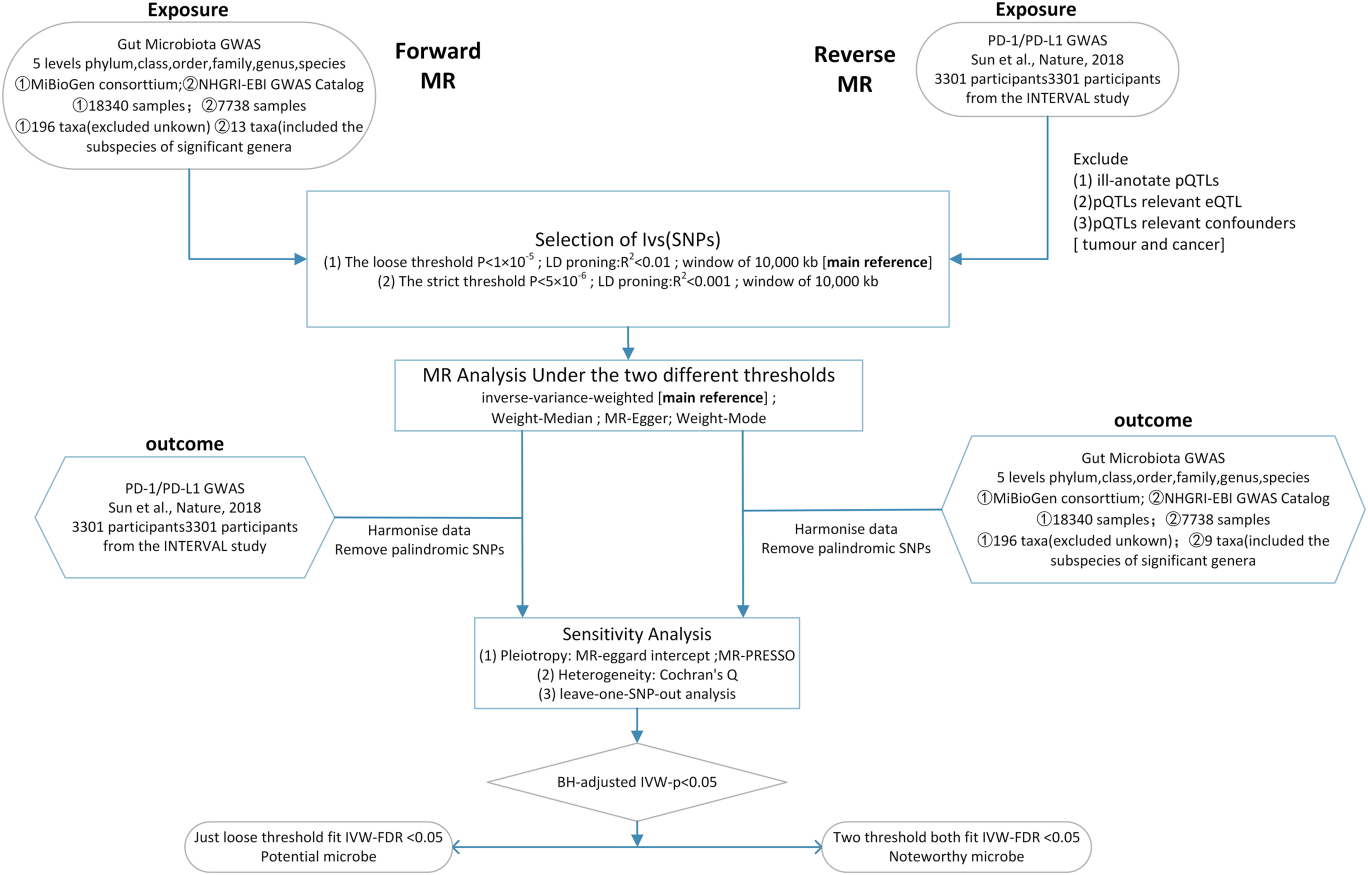
Workflow of this MR analysis. SNPs, single-nucleotide polymorphisms; MR, Mendelian randomization; LD, linkage disequilibrium; eQTL, expression quantitative trait loci; pQTL, protein quantitative trait loci.

Here, we conducted a two-sample bidirectional Mendelian randomization at two distinct thresholds to determine the causal relationship between PD-1/PD-L1 and the gut microbiota and explore potential biomarker microbes (**Figure 1**).

## Results

### Univariable forward MR

We used the loose threshold as the primary reference to investigate further potential linkages.

**Figure 2:**
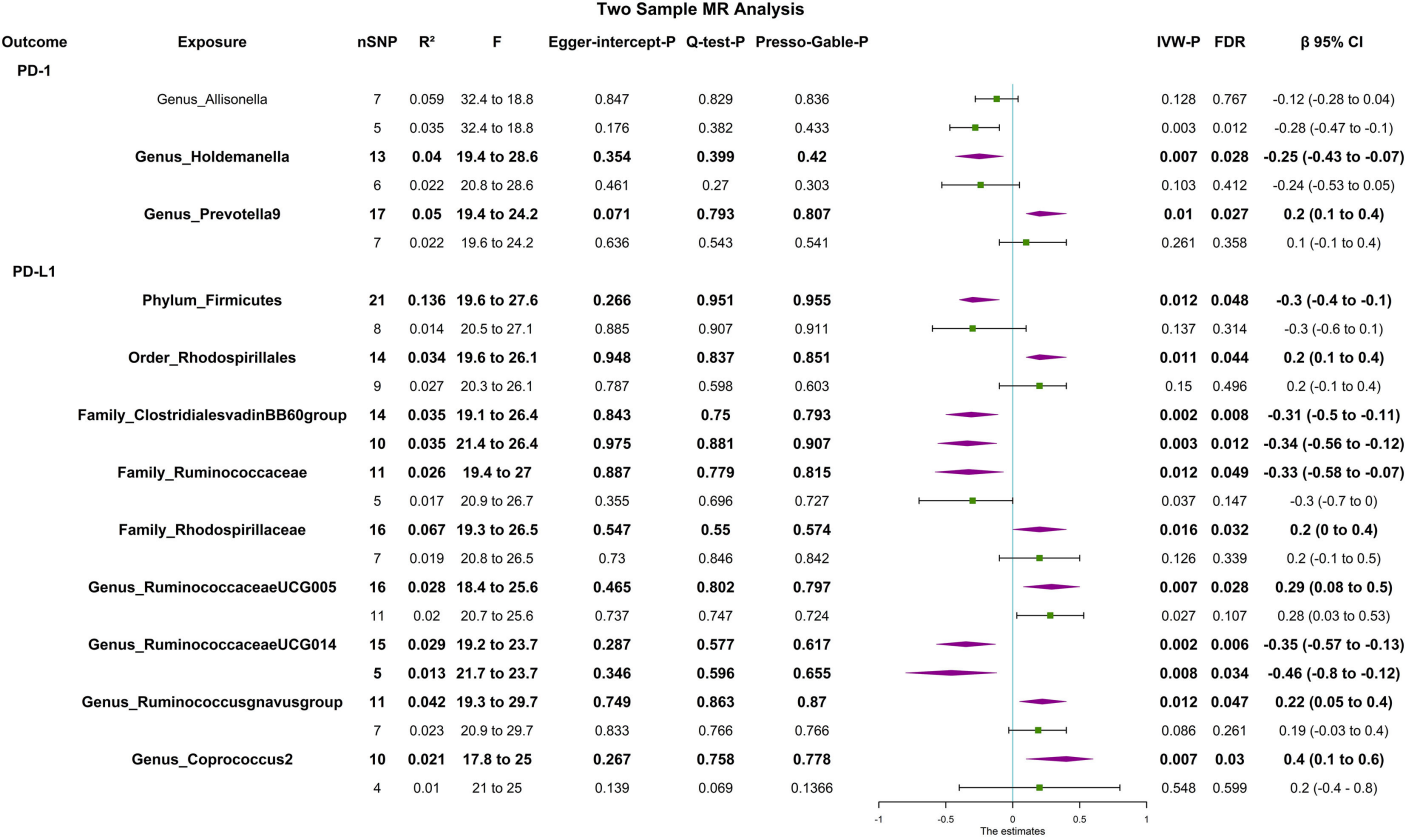
Forest plot of causal relationships estimated and sensitivity analysis for genus-level microbes and PD-1/PD-L1, the significant result (P_FDR_ <0.05) by the IVW method in forward two-sample MR analysis (includes two thresholds). The words in bold type indicate significant results. CI, confidence interval; F, F-statistics; R^2^, the genetic variants for instrument; IVW, inverse variance weighted.

The F statistic of any single genetic instrument that was used in the analysis was >10 to avoid weak instrument bias. In the forward MR with a relaxed threshold (P = 1 × 10^-5^, R^2^ = 0.01, LD = 10000), we discovered important microbes. For instance, genus_Holdemanella showed a negative correlation with PD-1 [βIVW = -0.25; 95% CI (-0.43 to -0.07); P_FDR_ = 0.028] and genus_Prevotella9 showed a positive correlation with PD-1 [βIVW = 0.2; 95% CI (0.1 to 0.4); P_FDR_ = 0.027]; order_Rhodospirillales [βIVW = 0.2; 95% CI (0.1 to 0.4); P_FDR_ = 0.044], family_Rhodospirillaceae [βIVW = 0.2; 95% CI (0 to 0.4); P_FDR_ = 0.032], genus_Ruminococcaceae_UCG005 [βIVW = 0.29; 95% CI (0.08 to 0.5); P_FDR_ = 0.028], genus_Ruminococcus_gnavus_group [βIVW = 0.22; 95% CI (0.05 to 0.4); P_FDR_ = 0.029], and genus_Coprococcus_2 [βIVW = 0.4; 95% CI (0.1 to 0.6); P_FDR_ = 0.018] were positively correlated with PD-L1; and phylum_Firmicutes [βIVW = -0.3; 95% CI (-0.4 to -0.1); P_FDR_ = 0.031], family_Clostridiales_vadin_BB60_group [βIVW = -0.31; 95% CI (-0.5 to -0.11), P_FDR_ = 0.008], family_Ruminococcaceae [βIVW = -0.33; 95% CI (-0.58 to -0.07); P_FDR_ = 0.049], and genus_Ruminococcaceae UCG014 [βIVW = -0.35; 95% CI (-0.57 to -0.13); P_FDR_ = 0.006] were negatively correlated with PD-L1. MR-PRESSO detected no horizontal pleiotropy (P _Presso Gable_>0.05). The weighted median and the weighted mode yielded similar patterns of effects or directions except for MR-Egger in some exposures, and the difference possibly due to the power of the MR-Egger method was smaller (11). According to Cochran’s Q statistic, there was no evidence of pleiotropy across instrument effects (Cochran’s Q_IVW_ >0.05). Analysis of MR-Egger intercepts revealed no indication of directional pleiotropy (P _Intercept >_0.05). The leave-one-out analysis identified all taxonomic groups exhibiting robustness under the loose threshold, except for genus_Prevotella_9 (**Figure 2**). More information is available in **Supplemental Table 1** (**Table S1**).

### Univariable reverse MR

In the reverse two-sample MR with the loose threshold (P = 1 × 10^-5^, R² = 0.01, window = 10,000), PD-1 was negatively related to genus_Terrisporobacter [βIVW = 0.2; 95% CI (0 to 0.3); P_FDR_ = 0.029], PD-L1 was positively related to family_Peptococcaceae [βIVW = 0.15; 95% CI (0.05 to 0.25); P_FDR_ = 0.019], and PD-L1 was negatively correlated with family_Porphyromonadaceae [βIVW = -0.1; 95% CI (-0.2 to 0); P_FDR_ = 0.017], genus_Odoribacter [βIVW = -0.1; 95% CI (-0.2 to 0); P_FDR_ = 0.022], and genus_Parabacteroides [βIVW = -0.1; 95% CI (-0.18 to -0.02); P_FDR_ = 0.042]. The sensitivity analysis showed no evidence of pleiotropy or heterogeneity. Other taxa in addition to the genera Parabacteroides and Odoribacter had robust results in the leave-one-out analysis (**Table 1**).

**Table 1:** Significant microbiota (IVW_FDR_<0.05) in reverse MR analysis.

Exposure	Outcome	F	Variants		IVW				Weighted Median		Weighted Mode		MR Egger			Cochran’s Q_IVW_	Presso Gable P
			nSNP	Outlier	βIVW 95%CI	P	P_FDR_	Beta	P	Beta	P	Beta	P	Beta	P _Intercept_		
**PD-L1**																	
	**Genus_Odoribacter**	19.6-37	5	1	-0.1 (-0.2 - 0)	0.005	0.022	-0.127	0.036	-0.132	0.183	-0.131	0.717	0.067	0.316	0.492	0.566
	**Genus_Parabacteroides**	19.6-37	6	0	-0.1 (-0.18 - -0.02)	0.010	0.042	-0.104	0.245	-0.062	0.597	-0.041	0.090	-0.317	0.196	0.335	0.391
	**Family_Peptococcaceae**	19.6-37	5	1	0.15 (0.05 - 0.25)	0.005	0.019	0.151	0.019	0.163	0.171	0.167	0.947	0.016	0.565	0.689	0.720
	**Family_Porphyromonadaceae**	19.6-37	6	0	-0.1 (-0.2 - 0)	0.007	0.017	-0.102	0.009	-0.124	0.099	-0.141	0.239	-0.192	0.535	0.823	0.837
**PD-1**																	
	**Genus_Barnesiella**	19.9-88	7	0	0 (-0.1 - 0.1)	0.430	0.440	0.037	0.376	0.051	0.364	0.092	0.440	0.268	0.497	0.200	0.228
		20.9-88	4	0	0.13 (0.03 - 0.23	0.014	0.028	0.127	0.059	0.119	0.317	0.099	0.412	0.425	0.543	0.804	0.824
	**Genus_LachnospiraceaeUCG001**	19.9-88	7	0	-0.1 (-0.2 - 0.1)	0.359	0.927	-0.052	0.793	-0.018	0.890	-0.016	0.927	0.039	0.830	0.162	0.188
		20.9-88	3	1	-0.2 (-0.3 - 0)	0.030	0.047	-0.157	0.035	-0.194	0.206	-0.231	0.470	0.537	0.387	0.351	–
	**Genus_Terrisporobacter**	19.9-88	6	1	0.2 (0 - 0.3)	0.015	0.029	0.151	0.013	0.177	0.162	0.205	0.962	0.523	0.377	0.918	0.929
		20.9-88	3	1	0.2 (0 - 0.3)	0.049	0.090	0.175	0.068	0.202	0.235	0.215	0.609	0.433	0.744	0.807	–
	**Genus_Veillonella**	19.9-88	7	0	-0.1 (-0.2 - 0)	0.128	0.406	-0.077	0.203	-0.085	0.413	-0.094	0.956	-0.021	0.883	0.353	0.373
		20.9-88	4	0	-0.17 (-0.29 - -0.04)	0.009	0.019	-0.167	0.054	-0.148	0.380	-0.110	0.419	-0.523	0.561	0.737	0.770

### The stability of validation MR

**Figure 3:**
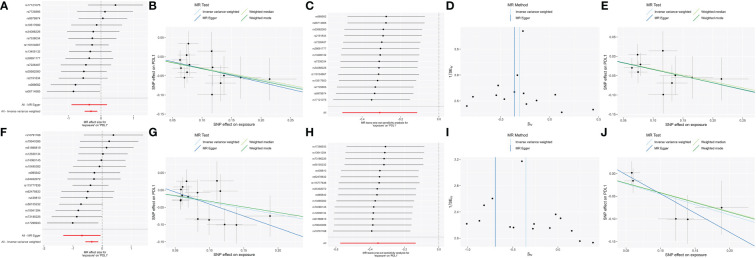
The significant (P_FDR_ <0.05) and robust results (Family_ClostridialesvadinBB60group and Genus_RuminococcaceaeUCG014) in forward MR analysis with two different thresholds. Scatter plot of microbe-related SNP effects on PD-L1, with the slope of each line corresponding to the estimated MR effect per method. Vertical and horizontal black lines around each point show the 95% confidence interval for each polymorphism exposure association and polymorphism outcome association. Forest plot lists single and combined (IVW and MR egger) SNP MR-estimated effect sizes; the effect estimates represent the β for PD-L1 per one-s.d. increase in mean microbes. The one-sided leave-one-out and symmetric funnel plots meant that the results were stable without outliers. Family_Clostridiales_vadin_BB60_group: **(A)** Forest plot at the loose threshold. **(B)** MR scatter at the loose threshold. **(C)** Leave one out at the loose threshold. **(D)** Funnel plot at the loose threshold. **(E)** MR scatter at the strict threshold. Genus_Ruminococcaceae_UCG014: **(F)** Forest plot at the loose threshold. **(G)** MR scatter at the loose threshold. **(H)** Leave one out at the loose threshold. **(I)** Funnel plot at the loose threshold. **(J)** MR scatter at the strict threshold.

To confirm the resulting stability, we conducted strict threshold analyses using the threshold (P = 5 × 10^−6^, R² = 0.001, window = 10,000) in bidirectional MR analyses. In forward MR, the family_ClostridialesvadinBB60 group [βIVW = -0.34 95% CI (-0.56 to -0.12); P_FDR_ = 0.012] and genus_RuminococcaceaeUCG014 [βIVW = -0.46; 95% CI (-0.8 to -0.12); P_FDR_ = 0.034] were negatively correlated with PD-L1, through both sensitivity analysis and leave-one-out analysis (**Figure 3**).

### Species-level MR

**Figure 4:**
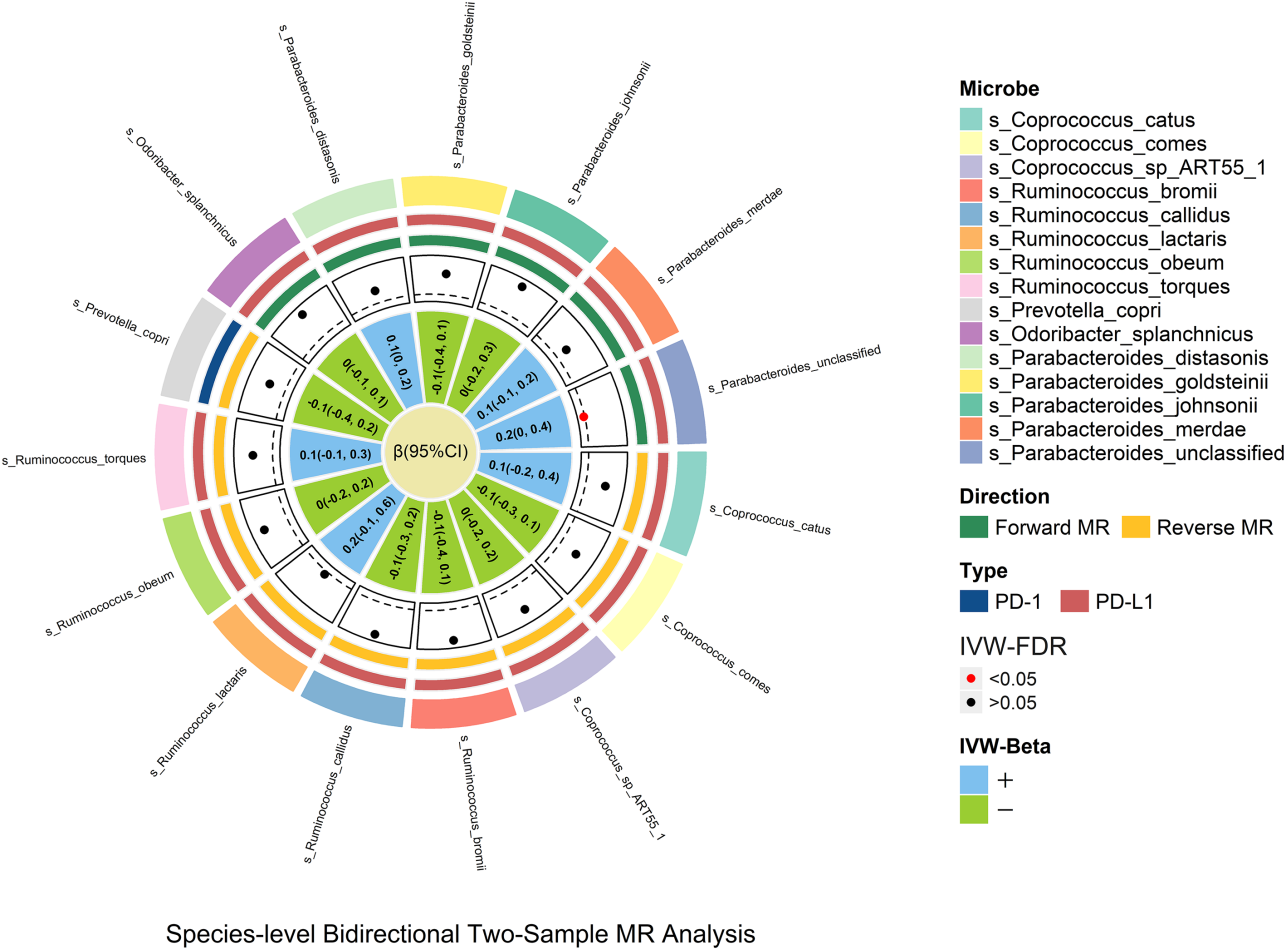
In the species-level bidirectional two-sample MR analysis, microbial features were prefixed with species(s). The selection of species-level microbes is based on the significant result of genus-level microbes. The MR estimates and 95% CI values are shown in the plot. The point of the plot indicates the P value of IVW, and red indicates significance (P_FDR_ <0.05). The “+” and “-” in the legend indicate the direction of the estimate effect (beta).

Species_Parabacteroides_unclassified was positively related to PD-L1 [βIVW = 0.2; 95% CI (0-0.4); P_FDR_ = 0.029], and no causal relationship was found between other genetically determined gut microbes. Species_Parabacteroides_unclassified was identified through sensitivity analysis but did not pass leave-one-out analysis (**Figure 4**).

## Discussion

In this work, we revealed associations between the relative abundance of the gut microbiota and the concentration of PD-1/PD-L1 by employing genetic variations as unconfounded proxies. To examine more potential associations and verify the outcome’s dependability, we utilized two alternative thresholds in two-sample bidirectional Mendelian randomization. Moreover, we conducted an expanding MR analysis based on the species-level dataset from another GWAS to explore potential relationships.

Phylum_Firmicutes is the largest of the four bacterial phyla (12), making up approximately 90% of the human genome. We discovered that phylum_Firmicutes reduced the blood’s PD-L1 level at the loose threshold. In the context of ICB therapy, phylum_Firmicutes was identified in a prior study as response-associated (13). Our investigation supports this causal connection. In our work, the family_Ruminococcaceae and family_Clostridiales_Vadin_BB60 group, members of phylum_Firmicutes, both led to decreased PD-L1 levels. A prior study found that family_Ruminococcaceae increased SCFA production (14), CD8 T-cell infiltration into the tumor microenvironment, and effectiveness of anti-PD-L1 therapy against colon cancer in mice (15). It exhibits beneficial effects in various races, malignancies, and geographic areas and has received substantial clinical validation [China; HCC (16);/America; melanoma (12);/America; melanoma (10);]. SCFA production seems to activate T cells against malignant cells and further decrease PD-L1. Famliy_Ruminococcaceae includes genus_Ruminococcaceae_UCG005, genus_Ruminococcaceae_UCG014, and genus_Ruminococcus_gnavus_group. Interestingly, they have varying effects on the PD-L1 level. Although not depicted in the prior investigation, genus_Ruminococcaceae_UCG014 has a detrimental effect on PD-L1 and is confirmed at two distinct thresholds. Genus_Ruminococcus_gnavus_group and genus_Ruminococcaceae_UCG005 exhibited a trend of a positive influence on PD-L1. The genus_Ruminococcus_gnavus group includes iso-bile acid-producing organisms. The iso-bile acid route detoxifies deoxycholic acid, causes DNA damage by the formation of free radicals, and has been linked to multiple cancers (17). It also favors the growth of the important genus_Bacteroides (18). *Bacteroides fragilis* has a positive association with PD-L1 expression and the PD-1 checkpoint pathway (19). Genus_Ruminococcaceae_UCG005 is abundant and deemed a biomarker taxon in HCC patients with hepatitis B or C virus infection (20) and lung adenocarcinoma patients (21). Genus_Coprococcus_2 is a subspecies of phylum_Firmicutes, and a prior study found that it was enriched in a high-fat-induced liver cancer model in male rats; it produces butyrate (14). SCFAs, such as butyrate or propionate, impact intestinal immunological homeostasis, affecting Tregs, γδ T cells, and effector T cells and participating in immunomodulatory and anti-inflammatory properties (5). However, its use in cancer immunotherapy remains contentious. Both positive and negative impacts are possible (5). Intriguingly, our study also shows that the SCFA producer may have a different effect on cancer, and these effects require further research. Despite the lack of pertinent analysis of family_Clostridiales_Vadin_BB60_group, two different thresholds support it as a protective factor against PD-L1, making it a possible target gut microbe for subsequent investigation. Genus_Holdemanella is a member of the family Erysipelotrichaceae; it produces SCFAs that, in humans, modulate intestinal immune homeostasis (5). Considering that it also lowered the expression of PD-1 in our study, it is possible that it affects PD-1 expression and further prevents immune escape in cancer cells and against cancer. Genus_Prevotella_9 is enriched in patients with advanced, unresectable hepatocellular carcinoma, according to a study (22). Curiously, another study revealed that genus_Prevotella_9 is deficient in bladder cancer tissue (23). Since genus_Prevotella_9 promoted PD-1 in our study, it may have various effects on different tumors, and its function requires further study. Studies on the relevance of order_ Rhodospirillales and family_Rhodospirillaceae to the human body are lacking, and additional investigation is required to investigate their potential relationship with the human immune system.

Early research shows that genus_Parabacteroides in colorectal cancer (CRC) (24) and early HCC versus cirrhosis (25) has the potential to become a biomarker, and we found that PD-L1 has a negative correlation with it. Genus_Odoribacter, a butyrate producer, has been found at lower levels in patients with breast cancer and rectal carcinoma (26), and it is essential in several related taxonomic models of CRC (27). Bacteroidetes (phylum) includes genus_Odoribacter and genus_Parabacteroides. Genus_Odoribacter is positively involved in non-ribosomal peptide structures and negatively involved in the metabolism of phenylalanine and cyanoamino acids. Family_Porphyromonadaceae has been identified as a potential biomarker for CRC recurrence and patient prognosis (28); case-control study results showed that healthy controls had a higher relative abundance of family_Porphyromonadaceae than primary liver cancer patients (29), and combined analysis with our study confirmed its potential as a biomarker in cancer. Family_ Peptococcaceae and genus_Terrisporobacter have been lacking in studies of the human immune system, so it is possible that we found microbes that can be applied as new biomarkers for PD-1/PD-L1 therapy or precancerous diagnosis.

In the species-level study, only species_Parabacteroides_unclassified demonstrated significant species-level verification results; it is possible that this is due to the lack of complete related species-level GWAS data and the variability of different region samples.

Overall, we demonstrated the potential causal relationships between microbiomes and PD-1/PD-L1. We have not found any similar research that has been published previously. This study expands the possibility of antineoplastic therapy and immunotherapy. We expanded the study to the species level, which enhanced the comprehensiveness of this study. We used two different thresholds in the MR analysis. First, the loose threshold widely used in the previous study allowed more potential microbes to be analyzed. Its significant result included many microbes considered as impact factors in a relevant ICB therapy study, which improves the credibility of this analysis. The strict threshold is approximated with the traditional MR threshold setting. Combining its result with the result of loose thresholds, we found two microbes that were never identified as impact factors for ICB therapy in a prior study. The newly identified microbiota will require further study. In reverse MR analysis, we investigated several microbes that can potentially be biomarkers in cancer therapy. Our study has a few limitations. First, while utilizing the largest single-cohort multiethnic GWAS to date, the sample size was quite limited and requires expansion, similar to prior research on the heritability of the microbiome (7, 30). Traditional GWAS and MR research frequently employ cohorts with hundreds of thousands of people, thereby enhancing power and lowering false associations; because of the low power and small sample sizes, many legitimate signals were unlikely to reach statistical significance at the study-wide level. Second, the GWAS we used to conduct MR combines many races, although the majority of individuals were of European descent (>72.3%). However, mixed races inevitably introduce bias into the results. Third, the heterogeneity of the makeup of the gut microbiota also results in a loss of strength. Similar to all research on the human gut microbiota, the sample variation is substantial, so its effect on the predicted heritability should not be overestimated.

## Method

**Figure 5:**
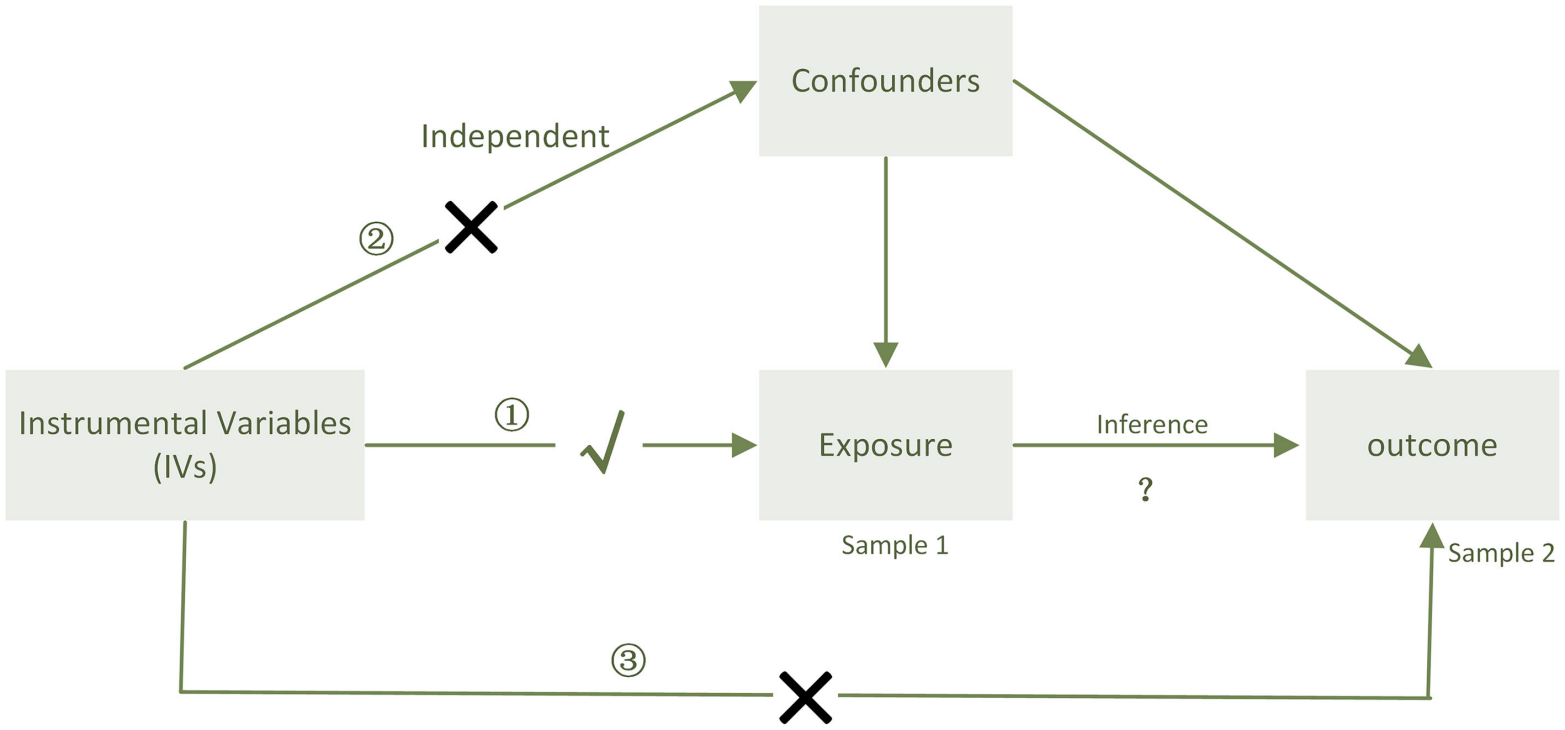
When sample 1(exposure) and sample 2(outcome) are used for causal estimates in MR inference, three assumptions must be satisfied (11). ① Relevance assumption: the genetic variations are highly related to the exposure, ② independence assumption: the genetic variants are not associated with any putative confounder of the association between exposure and result, and ③ exclusion restriction: the variants do not alter the outcome independently of exposure.

In our study, two-sample bidirectional MR analysis was undertaken at two distinct thresholds to determine the causal relationship between PD-1/PD-L1 and the gut microbiota. We utilized two distinct thresholds: one to explore the possibility of a relation and the other to validate the precision of the test (**Figure 5**).

### Data sources and methods

This study relied on publicly accessible summary-level data; ethical approval was acquired for all original investigations.

### Gut microbiota

Genetic variants of the gut microbiota were found in a large-scale association study involving 24 cohorts (18,340 participants) (7). Populations from Canada, the USA, Israel, South Korea, Denmark, Germany, the Netherlands, Belgium, Sweden, the UK, Finland, and Denmark were included in the cohorts. Twenty cohorts had samples of single ancestry, and most subjects (16 cohorts, N = 13,266) were of European ancestry. In 17 (n = 13,804) of the 24 cohorts, the participants’ mean ages ranged between 50 and 62. The microbiome quantitative trait locus (mbQTL) mapping study for each cohort only included taxa present in >10% of the samples, totaling 211 taxa (131 genera, 35 families, 20 orders, 16 classes, and 9 phyla). The study of binary trait locus mapping (mbQTL) covered the taxa that comprised 10%–90% of the included samples. There were 196 taxa included in our analysis (excluding 15 taxa that cannot be definitively classified and named) *Strain Categorization, the microbiota that we take into analysis (phylum-level to genus-level)* listed in **Supplemental Figure 5**.

For species-level analysis, we used another GWAS dataset that included 7,738 Dutch Microbiome Project (DMP) participants whose microbiota data were quality-controlled with LifeLines (30). A total of 58.1% of its members were women, and their ages ranged from 8 to 84 years (mean, 48.5 years). Data from 15 subordinate species taxa, whose genera were confirmed as significant (IVW_FDR_ <0.05) in the MiBioGen GWAS, were included in our study.

### PD-1 and PD-L1

We identified genetic predictors of cis-protein quantitative trait loci [cis-pQTLs] of PD-L1 based on summary statistics from the INTERVAL study, which recruited 3,301 healthy participants of European descent with an average age of 44 years and 48.9% women (31). Concerning trans-pQTLs, the functional genetic variations influence protein abundance with little or no attenuated effect on messenger RNA or ribosome levels (31, 32).

### Selection of the instrumental genetic factors

In the MR investigation of the link between the microbiota and PD1/PD-1, two thresholds were used to choose the IVs. For MR, the genetic variations that were representative of the microbiota trait were required to be sufficient; therefore, we decided on a locus-wide significance threshold P = 1 × 10^-5^ (7, 30), which was commonly utilized in prior microbe MR analyses, clustered for independence using PLINK in the two-sample MR tool (33) and the 1000 Genomes European data as the reference panel, using a looser cutoff of R^2^ < 0.01 and a window of 10,000-kb clumping. Another set of SNPs was fewer than the generally used threshold of 5 × 10^-6^ for emphasizing “suggestive” genetic variants (34) and clustering under the tighter cutoff of R^2^ < 0.001 and a 10,000-kb window. We supplemented the effect of allele frequency prior to clumping using data from the 3DSNP database (35). To avoid any confounding, we queried each SNP in the PhenoScanner database (36) for any past associations (P = 5 × 10^-8^) with probable confounders (that is, cancer and tumors).

In the MR investigation of the association between PD1/PD-1 and the microbiota, we selected pQTLs associated with genetically predicted PD-L1 or PD-1 from the INTERVAL study of the log-transformed relative fluorescence unit [log(RFU)] using the same threshold as described before. To limit random variability, only annotated pQTLs [i.e., those with proper identification and description (37)] from the RegulomeDB database were included. We selected cis-pQTLs by eliminating pQTLs that express quantitative trait loci (eQTLs) (32) from the RegulomeDB and VannoPortal databases (38, 39).

The effects of SNPs on exposure and outcome were then harmonized to ensure that the β values were signed for the identical alleles. After harmonizing the data, we eliminated SNPs with intermediate allele frequencies (>0.42). Radial-MR (40)and MR-PRSSO (41)were also performed to identify IVs with the best contribution to heterogeneity (alpha = 0.05/nSNP) and hence identify probable outliers. These outliers were removed from the IVs. Radial-MR was also utilized to determine whether the independence and exclusion restriction assumptions were violated. We eliminated the trait combination from the analysis for IVs < 3, and the MR analysis was conducted using the remaining SNPs.

### Testing instrument robustness and statistical validity

In the initial proteomic GWAS, it was found that the sentinel cis-pQTL explained 2% of the variation in circulating programmed death-1-ligand 2 levels (31). Using the web MR power calculation tool (42)(https://sb452.shinyapps.io/power/), when the causal effect achieves 0.345, there is 80% power if all detected cis-pQTLs explain 2% of the variation in PD-L1. The individual SNP effect size was estimated as the explainable variance with the formula [2 f(1 − f)β^2^], where f is the allele frequency and β is the regression coefficient (43). The formula [F statistic = beta2/se2] was used to calculate the F statistic (44). If the F statistic was ≥ 10, it implied a low probability of instrument bias in MR analysis (45).

### Statistical analysis

Cochran’s Q statistic was employed to assess the heterogeneity of the IVW meta-analysis; P < 0.10 indicates significant heterogeneity in the SNP effect estimates. When all IVs are valid instruments, the IVW method provides the most accurate estimate of the causal effect. However, because there are so few variants, the heterogeneity between the variant-specific estimates cannot be reliably estimated (11). Therefore, we conducted both random-effects IVW and fixed-effects IVW. When SNP >4 or without heterogeneity, we used random-effects IVW as the primary method; otherwise, we used fixed-effects IVW. The IVW method aggregated the Wald ratio estimates of each SNP into a single causal estimate for each risk factor, with each estimate derived by dividing the SNP–outcome association by the SNP–exposure association (46). The results of the IVW test with a P threshold corrected by FDR (P_FDR_) <0.05 are classified as significant. Considering that the FDR corrected by the number of microbes would be too stringent, we corrected the P threshold by the number of MR analysis methods. Since the IVW estimates can be biased if pleiotropic IVs are introduced, a series of sensitivity analyses were conducted to account for pleiotropy in the causal estimates. We examined the probable presence of horizontal pleiotropy using MR-Egger regression based on its intercept term, where the divergence from zero (P < 0.05) was interpreted as evidence of the presence of directional pleiotropic bias (47). In the presence of horizontal pleiotropy, the slope coefficient from MR-Egger regression provides a reliable estimate of the causal influence. As sensitivity analyses, we also conducted MR-Egger, weighted median, and weighted mode analyses based on varying hypotheses. Briefly, MR-Egger generally adheres to the Instrument Strength Independent of Direct Effect (InSIDE) and negligible measurement error (NOME) assumptions (47, 48). The weighted median method assumes that at least half of the instruments are valid (the weighted median method assumes the causal effect from the median of the weighted empirical density function of individual SNP effect estimates and permits up to 50% of information from variants to violate MR assumptions in the presence of horizontal pleiotropy) (49). The mode method is assumed to apply to the vast majority of genetic instruments (clusters the SNPs based on the similarity of causal effects and estimates the causal effect on the basis of the cluster with the most significant number of SNPs, thus providing an unbiased estimate if the SNPs contributing to the largest cluster are valid) (50). Leave-one-out analysis was performed to determine the impact of individual variations on the observed connections.

## Data availability statement

The original contributions presented in the study are included in the article/**Supplementary Materials**. Further inquiries can be directed to the corresponding author.

## Author contributions

Y-FH and G-XL conceived and designed the research. Y-FH analyzed the data and wrote the paper. W-MZ, Z-SW, HH, Q-YM, D-LS, LH, Y-YH and S-KN assisted in completing this research. All authors contributed to the article and approved the submitted version.
