# Supplementary material for: Causal relationships between gut microbiota and programmed cell death protein 1/programmed cell death-ligand 1: A bidirectional Mendelian randomization study
Source: Front Immunol. 2023 Mar 9;14:1136169. doi: 10.3389/fimmu.2023.1136169 (PMC10034163; doi:10.3389/fimmu.2023.1136169)
Supplement: Supplementary file 1 [file DataSheet_1.pdf]

Forward analysis on two different thresholds

Table S1. Significant result (any threshold IVW-FDR<0.05)

exposure	outcome	F	variants		R2	random IVW				Weighted median		MR Egger		Weight Mode		IVW Q	MR presso
			nSNP	outlier		βIVW 95% CI	P	P BH	beta	P	beta	P	beta	P	beta		Gable-p
PD-1																	
	phylum_Firmicutes	27.6-19.6	19	2	0.036	-0.2 (-0.4 to 0)"	0.097	0.387	-0.172	0.956	-0.009	0.340	0.223	0.820	0.043	0.455	0.454
		20.5-27.6	8	0	0.013	-0.42 (-0.78 to -0.06)"	0.022	0.056	-0.418	0.137	-0.376	0.793	0.221	0.343	-0.421	0.525	0.552
	class_Coriobacteriia	19.4-25	20	0	0.028	0.2 (0 to 0.4)	0.046	0.184	0.207	0.452	0.114	0.574	-0.145	0.796	0.049	0.703	0.709
		20.7-25	6	0	0.01	0 (-0.3 to 0.3)"	0.839	0.978	0.032	0.978	0.005	0.808	-0.078	0.966	-0.009	0.939	0.946
	order_Coriobacteriales	19.4-25	20	0	0.028	0.2 (0 to 0.4)	0.046	0.184	0.207	0.452	0.114	0.574	-0.145	0.796	0.049	0.703	0.709
		20.7-25	6	0	0.01	0 (-0.3 to 0.3)"	0.839	0.978	0.032	0.978	0.005	0.808	-0.078	0.966	-0.009	0.939	0.946
	family_Coriobacteriaceae	19.4-25	20	0	0.028	0.2 (0 to 0.4)	0.046	0.184	0.207	0.452	0.114	0.574	-0.145	0.796	0.049	0.703	0.709
		20.7-25	6	0	0.01	0 (-0.3 to 0.3)"	0.839	0.978	0.032	0.978	0.005	0.808	-0.078	0.966	-0.009	0.939	0.946
	genus_Allisonella	32.4-18.8	7	2	0.059	-0.12 (-0.28 to 0.04)"	0.128	0.767	-0.122	0.490	-0.070	0.690	-0.234	0.819	-0.034	0.829	0.836
		32.4-18.8	5	0	0.035	-0.28 (-0.47 to -0.1)	0.003	0.012	-0.282	0.030	-0.256	0.284	0.821	0.140	-0.278	0.382	0.433
	genus_Blautia	18.6-25.3	12	1	0.019	-0.04 (-0.29 to 0.21)"	0.773	0.939	-0.037	0.939	0.013	0.867	-0.047	0.820	0.049	0.492	0.540
		20.9-25.3	5	1	0.009	-0.44 (-0.86 to -0.03)	0.037	0.147	-0.442	0.117	-0.417	0.949	-0.046	0.316	-0.470	0.402	0.430
	genus_Slackia	19.8-25	9	0	0.022	-0.21 (-0.41 to -0.02)"	0.032	0.128	-0.213	0.093	-0.216	0.839	0.088	0.231	-0.251	0.706	0.716
		20.5-25	6	0	0.024	-0.24 (-0.47 to -0.01)"	0.045	0.180	-0.237	0.160	-0.222	0.984	0.012	0.343	-0.228	0.442	0.475
	genus_Barnesiella	19-25	17	0	0.024	0.22 (0.01 to 0.43)"	0.037	0.147	0.221	0.352	0.134	0.427	0.312	0.534	0.154	0.544	0.566
		20.8-25	11	0	0.013	0.2 (0 to 0.5)"	0.076	0.190	0.228	0.361	0.162	0.465	0.319	0.497	0.194	0.677	0.692
	genus_Clostridiumsensustricto1	17.3-22.1	9	0	0.021	-0.25 (-0.5 to 0)"	0.049	0.200	-0.252	0.188	-0.230	0.689	-0.120	0.385	-0.215	0.765	0.739
		20-22.1	6	0	0.011	-0.23 (-0.52 to 0.06)"	0.125	0.499	-0.228	0.346	-0.189	0.832	-0.071	0.812	-0.058	0.502	0.511
	genus_Holdemanella	19.4-28.6	13	0	0.04	-0.25 (-0.43 to -0.07)"	0.007	0.028	-0.247	0.143	-0.182	0.715	0.162	0.980	0.006	0.399	0.420
		20.8-28.6	6	0	0.022	-0.24 (-0.53 to 0.05)"	0.103	0.412	-0.242	0.642	-0.083	0.407	-1.980	0.852	0.053	0.270	0.303
	genus_RikenellaceaeRC9gutgroup	19-27.8	9	2	0.034	-0.17 (-0.31 to -0.02)"	0.022	0.090	-0.169	0.090	-0.170	0.586	-0.291	0.351	-0.163	0.792	0.800
		20.6-27.8	5	1	0.034	-0.16 (-0.35 to 0.03)"	0.094	0.266	-0.163	0.133	-0.193	0.929	-0.065	0.307	-0.210	0.516	0.544
	genus_Bifidobacterium	19.9-88.4	15	1	0.033	0.2 (0 to 0.5)"	0.017	0.067	0.249	0.354	0.144	0.354	-0.297	0.837	0.041	0.447	0.457
		20.8-88.4	8	0	0.021	0 (-0.3 to 0.3)"	0.988	0.989	0.002	0.936	-0.013	0.781	-0.093	0.989	-0.003	0.971	0.979
	genus_Paraprevotella	20.3-26.9	12	1	0.038	0.2 (0 to 0.4)"	0.049	0.122	0.176	0.491	0.087	0.514	-0.195	0.748	0.054	0.394	0.435
		21-26.9	9	0	0.027	0.1 (-0.1 to 0.3)"	0.240	0.599	0.115	0.524	0.082	0.864	-0.055	0.732	0.063	0.724	0.744
	genus_Prevotella9	19.4-24.2	17	2	0.05	0.2 (0.1 to 0.4)	0.010	0.027	0.218	0.033	0.255	0.014	0.610	0.128	0.266	0.793	0.807
		19.6-24.2	7	0	0.022	0.1 (-0.1 to 0.4)	0.261	0.358	0.131	0.171	0.205	0.392	0.254	0.269	0.233	0.543	0.541
	genus_Ruminococcus2	19.1-24.2	14	1	0.031	0.2 (0 to 0.5)"	0.040	0.161	0.242	0.194	0.207	0.209	0.409	0.252	0.298	0.907	0.919
		20.3-24.2	7	0	0.02	0.1 (-0.2 to 0.4)"	0.338	0.470	0.147	0.355	0.178	0.470	0.273	0.431	0.208	0.948	0.950
PD-L1																	
	phylum_Firmicutes	19.6-27.6	21	0	0.014	-0.3 (-0.4 to -0.1)	0.012	0.048	-0.250	0.177	-0.190	0.939	-0.018	0.445	-0.146	0.951	0.955
		20.5-27.1	8	0	0.014	-0.3 (-0.6 to 0.1)"	0.137	0.314	-0.272	0.228	-0.288	0.855	-0.154	0.235	-0.484	0.907	0.911
	phylum_Tenericutes	19-25	12	0	0.023	0.26 (0.04 to 0.49)"	0.022	0.086	0.265	0.303	0.164	0.871	0.062	0.670	0.104	0.388	0.420
		19.2-25	7	0	0.015	0.37 (0.07 to 0.67)"	0.017	0.067	0.367	0.074	0.341	0.954	-0.026	0.604	0.143	0.275	0.331
	class_Mollicutes	19-28.7	12	0	0.023	0.26 (0.04 to 0.49)"	0.022	0.086	0.265	0.303	0.164	0.871	0.062	0.670	0.104	0.388	0.420
		20.6-28.7	7	0	0.015	0.37 (0.07 to 0.67)"	0.017	0.067	0.367	0.074	0.341	0.954	-0.026	0.604	0.143	0.275	0.331
	class_Methanobacteria	19.3-26.8	10	2	0.067	0.2 (0 to 0.3)	0.030	0.121	0.152	0.113	0.138	0.249	0.343	0.322	0.137	0.913	0.921
		23.3-26.8	4	0	0.03	0.1 (-0.1 to 0.3)"	0.200	0.428	0.137	0.257	0.144	0.569	0.272	0.377	0.170	0.981	0.983
	order_Bacillales	18.9-26.7	11	0	0.078	-0.11 (-0.24 to 0.02)"	0.105	0.390	-0.107	0.195	-0.115	0.678	-0.129	0.468	-0.102	0.782	0.791
		20.6-28.7	5	0	0.03	-0.2 (-0.38 to -0.01)	0.035	0.088	-0.199	0.110	-0.194	0.855	0.076	0.381	-0.157	0.847	0.855
	order_Methanobacteriales	19.3-26.8	10	2	0.067	0.2 (0 to 0.3)"	0.030	0.076	0.152	0.113	0.138	0.249	0.343	0.322	0.137	0.913	0.921
		23.3-26.8	4	0	0.03	0.1 (-0.1 to 0.3)"	0.200	0.428	0.137	0.257	0.144	0.569	0.272	0.377	0.170	0.981	0.983
	order_MollicutesRF9	18.2-22.8	13	2	0.036	0.2 (0 to 0.4)"	0.037	0.148	0.216	0.111	0.236	0.444	0.235	0.333	0.215	0.514	0.553
		21.8-22.8	3	0	0.008	0.2 (-0.1 to 0.6)"	0.255	0.637	0.203	0.516	0.136	0.904	-0.056	0.668	0.116	0.623	
	order_Rhodospirillales	19.6-26.1	14	0	0.034	0.2 (0.1 to 0.4)"	0.011	0.044	0.237	0.022	0.301	0.632	0.209	0.174	0.312	0.837	0.851
		20.3-26.1	9	0	0.027	0.2 (-0.1 to 0.4)"	0.150	0.496	0.167	0.248	0.182	0.610	0.344	0.557	0.143	0.598	0.603
	family_ClostridialesvadinBB60group	19.1-26.4	14	2	0.035	-0.31 (-0.5 to -0.11)"	0.002	0.008	-0.308	0.029	-0.296	0.219	-0.361	0.138	-0.316	0.750	0.793
		21.4-26.4	10	1	0.035	-0.34 (-0.56 to -0.12)"	0.003	0.012	-0.340	0.029	-0.326	0.287	-0.349	0.121	-0.343	0.881	0.907
	family_Ruminococcaceae	19.4-27	11	0	0.026	-0.33 (-0.58 to -0.07)"	0.012	0.049	-0.326	0.101	-0.291	0.335	-0.289	0.327	-0.215	0.779	0.815
		20.9-26.7	5	0	0.017	-0.3 (-0.7 to 0)"	0.037	0.147	-0.338	0.117	-0.312	0.721	0.206	0.397	-0.225	0.696	0.727
	family_Methanobacteriaceae	19.3-26.8	10	2	0.067	0.2 (0 to 0.3)"	0.030	0.076	0.152	0.113	0.138	0.249	0.343	0.322	0.137	0.913	0.921
		23.3-26.8	4	0	0.03	0.1 (-0.1 to 0.3)"	0.200	0.428	0.137	0.257	0.144	0.569	0.272	0.377	0.170	0.981	0.983
	family_Rhodospirillaceae	19.3-26.5	16	0	0.067	0.2 (0 to 0.4)"	0.016	0.032	0.210	0.010	0.314	0.282	0.455	0.102	0.415	0.550	0.574
		20.8-26.5	7	0	0.019	0.2 (-0.1 to 0.5)"	0.126	0.339	0.198	0.170	0.231	0.567	0.481	0.269	0.296	0.846	0.842
	genus_RikenellaceaeRC9gutgroup	19-27.8	11	0	0.079	-0.05 (-0.18 to 0.08)"	0.463	0.661	-0.049	0.821	0.021	0.471	-0.319	0.496	0.119	0.470	0.477
		20.6-27.8	6	0	0.034	-0.2 (-0.4 to 0)"	0.037	0.083	-0.186	0.041	-0.224	0.586	-0.315	0.182	-0.277	0.560	0.588
	genus_RuminococcaceaeUCG005	18.4-25.6	16	0	0.028	0.29 (0.08 to 0.5)	0.007	0.028	0.290	0.098	0.244	0.786	0.082	0.964	-0.012	0.802	0.797
		20.7-25.6	11	1	0.02	0.28 (0.03 to 0.53)	0.027	0.107	0.280	0.203	0.228	0.590	0.178	0.943	-0.024	0.747	0.724
	genus_RuminococcaceaeUCG014	19.2-23.7	15	3	0.029	-0.35 (-0.57 to -0.13)	0.002	0.006	-0.354	0.039	-0.325	0.053	-0.694	0.226	-0.324	0.577	0.617
		21.7-23.7	5	1	0.013	-0.46 (-0.8 to -0.12)	0.008	0.034	-0.456	0.068	-0.419	0.118	-0.847	0.241	-0.427	0.596	0.655
	genus_Tyzzerella3	19.6-35.4	13	1	0.0712	-0.12 (-0.26 to 0.03)	0.112	0.448	-0.117	0.674	-0.041	0.933	-0.041	0.880	-0.026	0.703	0.717
		20.1-35.4	9	1	0.054	-0.17 (-0.34 to 0)	0.044	0.088	-0.174	0.088	-0.193	0.691	-0.266	0.287	-0.204	0.516	0.547
	genus_Veillonella	19.9-23.2	10	0	0.029	0.23 (0 to 0.45)	0.047	0.189	0.227	0.222	0.183	0.715	-0.235	0.468	0.199	0.630	0.644
		20.1-23.2	5	0	0.014	0.3 (0 to 0.7)"	0.060	0.144	0.323	0.087	0.378	0.683	1.706	0.203	0.530	0.338	0.389
	genus_Victivallis	18.9-25.3	12	0	0.08	0.07 (-0.05 to 0.19)	0.264	0.782	0.070	0.434	0.068	0.804	0.095	0.587	0.082	0.670	0.681
		21.5-25.3	6	0	0.05	0.18 (0.01 to 0.35)	0.035	0.139	0.180	0.120	0.170	0.764	-0.158	0.409	0.142	0.733	0.749
	genus_Ruminococcusgnavusgroup	19.3-29.7	11	1	0.042	0.22 (0.05 to 0.4)	0.012	0.047	0.224	0.038	0.246	0.814	0.096	0.152	0.331	0.863	0.870
		20.9-29.7	7	0	0.023	0.19 (-0.03 to 0.4)"	0.086	0.261	0.185	0.131	0.213	0.863	0.084	0.319	0.226	0.766	0.766
	genus_Coprococcus2	17.8-25	10	1	0.021	0.4 (0.1 to 0.6)"	0.007	0.03	0.355	0.047	0.359	0.499	-0.538	0.190	0.403	0.758	0.778
		21-25	7	0	0.01	0.2 (0 to 0.4)"	0.086	0.599	0.185	0.131	0.213	0.863	0.084	0.319	0.226	0.069	0.133

Table S2. The SNP related to confounders of PhenoScan

taxa	rsid	hg19_coordinates	a1	a2	trait	p
family_Veillonellaceae	rs79535861	chr13:38326692	A	C	Self-reported malignant melanoma	4.67E-06
family_Veillonellaceae	rs7324328	chr13:38330047	T	C	Self-reported malignant melanoma	7.03E-06
family_Veillonellaceae	rs11838712	chr13:38330756	A	G	Self-reported malignant melanoma	6.98E-06
family_Veillonellaceae	rs17056612	chr13:38344859	A	G	Self-reported malignant melanoma	6.37E-06
family_Veillonellaceae	rs17263892	chr13:38345556	A	C	Self-reported malignant melanoma	6.01E-06
family_Bifidobacteriaceae	rs6730157	chr2:135907088	A	G	Melanoma skin cancer	4.82E-06
family_Bifidobacteriaceae	rs1561277	chr2:136092061	A	C	Melanoma skin cancer	5.21E-07
family_Bifidobacteriaceae	rs1446585	chr2:136407479	A	G	Melanoma skin cancer	3.27E-06

family_Bifidobacteriaceae	rs2011946	chr2:136817616	A	C	Melanoma skin cancer	6.88E-07
family_Bifidobacteriaceae	rs932206	chr2:136825272	T	C	Melanoma skin cancer	2.68E-07
phylum_Actinobacteria	rs6730157	chr2:135907088	A	G	Melanoma skin cancer	4.82E-06
phylum_Actinobacteria	rs1561277	chr2:136092061	A	C	Melanoma skin cancer	5.21E-07
phylum_Actinobacteria	rs1446585	chr2:136407479	A	G	Melanoma skin cancer	3.27E-06
phylum_Actinobacteria	rs2011946	chr2:136817616	A	C	Melanoma skin cancer	6.88E-07
phylum_Actinobacteria	rs932206	chr2:136825272	T	C	Melanoma skin cancer	2.68E-07
class_Actinobacteria	rs6730157	chr2:135907088	A	G	Melanoma skin cancer	4.82E-06
class_Actinobacteria	rs1561277	chr2:136092061	A	C	Melanoma skin cancer	5.21E-07
class_Actinobacteria	rs1446585	chr2:136407479	A	G	Melanoma skin cancer	3.27E-06
class_Actinobacteria	rs2011946	chr2:136817616	A	C	Melanoma skin cancer	6.88E-07
class_Actinobacteria	rs932206	chr2:136825272	T	C	Melanoma skin cancer	2.68E-07
order_Bifidobacteriales	rs6730157	chr2:135907088	A	G	Melanoma skin cancer	4.82E-06
order_Bifidobacteriales	rs1561277	chr2:136092061	A	C	Melanoma skin cancer	5.21E-07
order_Bifidobacteriales	rs1446585	chr2:136407479	A	G	Melanoma skin cancer	3.27E-06
order_Bifidobacteriales	rs2011946	chr2:136817616	A	C	Melanoma skin cancer	6.88E-07
order_Bifidobacteriales	rs932206	chr2:136825272	T	C	Melanoma skin cancer	2.68E-07
genus_Oxalobacter	rs12925026	chr16:89792856	T	C	Self-reported malignant melanoma	6.13E-32

Table S3. the selected micobiota SNP which taking into analysis

the *Forward* Two-sample-MR with the loose threshold ($P=1\times10^{-5}$,$R^2=0.01$,window=10000)

Exposure	rsid	effect_allele	other_allele	beta	eaf	chr	se	pval	samplesize	F
phylum_Firmicutes	rs10164700	A	G	0.082	0.155	2	0.018	4.85E-06	17817	20.5
phylum_Firmicutes	rs10169367	T	C	0.092	0.356	2	0.018	2.78E-06	18340	26.4
phylum_Firmicutes	rs10183008	A	G	0.091	0.363	2	0.018	5.60E-06	18339	25.3
phylum_Firmicutes	rs10189248	A	G	0.089	0.351	2	0.018	5.81E-06	18340	24.6
phylum_Firmicutes	rs10201035	T	C	0.090	0.351	2	0.018	4.77E-06	18340	23.9
phylum_Firmicutes	rs10209007	G	C	0.089	0.153	2	0.018	7.40E-07	17812	24.0
phylum_Firmicutes	rs10933355	A	G	0.080	0.155	2	0.018	8.50E-06	17818	19.5
phylum_Firmicutes	rs11108755	A	T	0.050	0.353	12	0.011	4.31E-06	17859	21.2
phylum_Firmicutes	rs112334273	G	A	0.063	0.136	21	0.013	9.26E-07	18217	24.3
phylum_Firmicutes	rs113037048	T	C	0.082	0.149	2	0.018	4.83E-06	17817	20.6
phylum_Firmicutes	rs11683620	G	A	0.084	0.156	2	0.018	3.31E-06	17816	21.5
phylum_Firmicutes	rs11689387	G	A	0.082	0.153	2	0.018	5.46E-06	17817	20.3
phylum_Firmicutes	rs11690370	T	C	0.083	0.155	2	0.018	4.83E-06	17817	20.6
phylum_Firmicutes	rs12554342	G	C	-0.053	0.231	9	0.012	6.81E-06	18332	20.2
phylum_Firmicutes	rs12983973	A	G	-0.069	0.217	19	0.016	7.95E-06	18337	19.3
phylum_Firmicutes	rs13386595	G	A	0.085	0.157	2	0.018	2.29E-06	17813	21.8
phylum_Firmicutes	rs13401441	T	G	0.097	0.330	2	0.019	4.38E-06	17859	27.1
phylum_Firmicutes	rs13409228	A	G	0.095	0.330	2	0.019	8.45E-06	17859	25.6
phylum_Firmicutes	rs13411251	G	T	0.083	0.154	2	0.018	4.07E-06	17817	21.0
phylum_Firmicutes	rs13421739	C	G	0.094	0.361	2	0.018	1.95E-06	18340	27.6
phylum_Firmicutes	rs1374597	T	G	0.093	0.356	2	0.018	3.34E-06	18340	26.4
phylum_Firmicutes	rs1568247	A	G	0.080	0.152	2	0.018	8.39E-06	17817	19.5
phylum_Firmicutes	rs1963579	A	T	-0.068	0.213	19	0.016	9.68E-06	18340	18.7
phylum_Firmicutes	rs1984783	G	A	-0.068	0.213	19	0.016	9.84E-06	18340	18.6
phylum_Firmicutes	rs2009919	T	C	0.054	0.446	17	0.011	4.95E-07	17859	25.5
phylum_Firmicutes	rs2220596	G	A	-0.070	0.213	19	0.016	6.12E-06	18340	19.6
phylum_Firmicutes	rs2273429	A	G	-0.070	0.290	14	0.015	9.26E-06	18337	21.0
phylum_Firmicutes	rs2332027	G	A	-0.048	0.416	4	0.010	4.05E-06	18340	21.2
phylum_Firmicutes	rs2547978	A	G	0.047	0.391	5	0.011	8.57E-06	18338	19.7
phylum_Firmicutes	rs28651047	T	C	0.083	0.154	2	0.018	4.34E-06	17817	20.9
phylum_Firmicutes	rs28814056	C	G	0.086	0.155	2	0.018	1.67E-06	17814	22.5
phylum_Firmicutes	rs35393748	A	T	-0.068	0.213	19	0.016	9.97E-06	18340	18.7
phylum_Firmicutes	rs36111799	C	T	-0.048	0.452	10	0.011	5.17E-06	18226	20.8
phylum_Firmicutes	rs3792064	G	A	0.090	0.153	2	0.018	6.75E-07	17812	24.2
phylum_Firmicutes	rs3792068	G	A	0.087	0.155	2	0.018	1.36E-06	17812	22.8
phylum_Firmicutes	rs3852931	C	T	-0.048	0.430	20	0.010	4.53E-06	18260	21.1
phylum_Firmicutes	rs3931771	G	A	-0.069	0.217	19	0.016	9.51E-06	18336	19.2
phylum_Firmicutes	rs4289208	C	T	0.091	0.358	2	0.018	4.99E-06	18339	25.8
phylum_Firmicutes	rs4750583	A	G	0.062	0.057	10	0.014	5.79E-06	16934	19.9
phylum_Firmicutes	rs4933026	T	A	-0.069	0.214	19	0.016	8.43E-06	18336	19.2
phylum_Firmicutes	rs56199908	T	C	-0.186	0.077	9	0.041	8.67E-06	5618	20.6
phylum_Firmicutes	rs58181266	A	G	0.083	0.158	2	0.018	3.71E-06	17817	20.9
phylum_Firmicutes	rs58880850	C	A	-0.069	0.214	19	0.016	8.90E-06	18340	18.9
phylum_Firmicutes	rs6436973	G	C	0.083	0.156	2	0.018	4.28E-06	17817	20.8
phylum_Firmicutes	rs6436974	A	G	0.083	0.151	2	0.018	3.57E-06	17817	21.1
phylum_Firmicutes	rs6711453	G	A	0.082	0.152	2	0.018	5.55E-06	17817	20.4
phylum_Firmicutes	rs6752031	T	C	0.082	0.152	2	0.018	6.16E-06	17817	20.2
phylum_Firmicutes	rs6814436	T	C	0.068	0.137	4	0.015	6.80E-06	18336	20.3
phylum_Firmicutes	rs6815608	T	C	0.094	0.243	4	0.021	7.24E-06	16731	19.7
phylum_Firmicutes	rs7247191	T	C	-0.071	0.217	19	0.016	4.73E-06	18340	20.5
phylum_Firmicutes	rs72738886	T	C	0.086	0.030	5	0.019	7.68E-06	16934	20.6
phylum_Firmicutes	rs72771021	C	T	-0.141	0.034	10	0.031	5.12E-06	9068	21.0
phylum_Firmicutes	rs74905079	G	A	0.081	0.152	2	0.018	6.46E-06	17816	20.2
phylum_Firmicutes	rs7557870	T	A	0.099	0.338	2	0.019	3.26E-06	17859	27.8
phylum_Firmicutes	rs7573799	C	G	-0.070	0.165	2	0.015	8.05E-06	17859	20.6
phylum_Firmicutes	rs7593057	G	C	0.081	0.155	2	0.018	7.38E-06	17817	19.8
phylum_Firmicutes	rs7600092	T	C	0.098	0.338	2	0.019	4.07E-06	17859	27.4
phylum_Firmicutes	rs7601602	G	A	0.082	0.155	2	0.018	5.56E-06	17815	20.5
phylum_Firmicutes	rs7975768	C	G	0.051	0.349	12	0.011	2.82E-06	17859	22.0
phylum_Firmicutes	rs8085381	G	A	0.065	0.251	18	0.015	8.67E-06	18339	19.6
phylum_Firmicutes	rs8112095	A	G	-0.069	0.217	19	0.016	9.75E-06	18337	19.2
phylum_Firmicutes	rs9730854	G	A	-0.048	0.405	10	0.011	9.26E-06	17745	19.7
phylum_Firmicutes	rs978023	A	T	0.094	0.331	2	0.019	9.68E-06	17859	25.2

phylum_Firmicutes	rs2547978	A	G	0.047	0.391	5	0.011	8.57E-06	18338	19.7
phylum_Firmicutes	rs28651047	T	C	0.083	0.154	2	0.018	4.34E-06	17817	20.9
phylum_Firmicutes	rs28814056	C	G	0.086	0.155	2	0.018	1.67E-06	17814	22.5
phylum_Firmicutes	rs35393748	A	T	-0.068	0.213	19	0.016	9.97E-06	18340	18.7
phylum_Firmicutes	rs36111799	C	T	-0.048	0.452	10	0.011	5.17E-06	18226	20.8
phylum_Firmicutes	rs3792064	G	A	0.090	0.153	2	0.018	6.75E-07	17812	24.2
phylum_Firmicutes	rs3792068	G	A	0.087	0.155	2	0.018	1.36E-06	17812	22.8
phylum_Firmicutes	rs3852931	C	T	-0.048	0.430	20	0.010	4.53E-06	18260	21.1
phylum_Firmicutes	rs3931771	G	A	-0.069	0.217	19	0.016	9.51E-06	18336	19.2
phylum_Firmicutes	rs4289208	C	T	0.091	0.358	2	0.018	4.99E-06	18339	25.8
phylum_Firmicutes	rs4750583	A	G	0.062	0.057	10	0.014	5.79E-06	16934	19.9
phylum_Firmicutes	rs4933026	T	A	-0.069	0.214	19	0.016	8.43E-06	18336	19.2
phylum_Firmicutes	rs56199908	T	C	-0.186	0.077	9	0.041	8.67E-06	5618	20.6
phylum_Firmicutes	rs58181266	A	G	0.083	0.158	2	0.018	3.71E-06	17817	20.9
phylum_Firmicutes	rs58880850	C	A	-0.069	0.214	19	0.016	8.90E-06	18340	18.9
phylum_Firmicutes	rs6436973	G	C	0.083	0.156	2	0.018	4.28E-06	17817	20.8
phylum_Firmicutes	rs6436974	A	G	0.083	0.151	2	0.018	3.57E-06	17817	21.1
phylum_Firmicutes	rs6711453	G	A	0.082	0.152	2	0.018	5.55E-06	17817	20.4
phylum_Firmicutes	rs6752031	T	C	0.082	0.152	2	0.018	6.16E-06	17817	20.2
phylum_Firmicutes	rs6814436	T	C	0.068	0.137	4	0.015	6.80E-06	18336	20.3
phylum_Firmicutes	rs6815608	T	C	0.094	0.243	4	0.021	7.24E-06	16731	19.7
phylum_Firmicutes	rs7247191	T	C	-0.071	0.217	19	0.016	4.73E-06	18340	20.5
phylum_Firmicutes	rs72738886	T	C	0.086	0.030	5	0.019	7.68E-06	16934	20.6
phylum_Firmicutes	rs72771021	C	T	-0.141	0.034	10	0.031	5.12E-06	9068	21.0
phylum_Firmicutes	rs74905079	G	A	0.081	0.152	2	0.018	6.46E-06	17816	20.2
phylum_Firmicutes	rs7557870	T	A	0.099	0.338	2	0.019	3.26E-06	17859	27.8
phylum_Firmicutes	rs7573799	C	G	-0.070	0.165	2	0.015	8.05E-06	17859	20.6
phylum_Firmicutes	rs7593057	G	C	0.081	0.155	2	0.018	7.38E-06	17817	19.8
phylum_Firmicutes	rs7600092	T	C	0.098	0.338	2	0.019	4.07E-06	17859	27.4
phylum_Firmicutes	rs7601602	G	A	0.082	0.155	2	0.018	5.56E-06	17815	20.5
phylum_Firmicutes	rs7975768	C	G	0.051	0.349	12	0.011	2.82E-06	17859	22.0
phylum_Firmicutes	rs8085381	G	A	0.065	0.251	18	0.015	8.67E-06	18339	19.6
phylum_Firmicutes	rs8112095	A	G	-0.069	0.217	19	0.016	9.75E-06	18337	19.2
phylum_Firmicutes	rs9730854	G	A	-0.048	0.405	10	0.011	9.26E-06	17745	19.7
phylum_Firmicutes	rs978023	A	T	0.094	0.331	2	0.019	9.68E-06	17859	25.2
phylum_Firmicutes	rs992074	C	T	0.233	0.021	21	0.051	8.52E-06	3501	21.0
phylum_Firmicutes	rs9973480	C	A	0.082	0.154	2	0.018	6.82E-06	17816	20.1
order_Rhodospirillales	rs1035406	G	A	-0.115	0.191	5	0.025	4.07E-06	8585	21.4
order_Rhodospirillales	rs11591293	G	T	0.072	0.163	10	0.016	4.69E-06	8602	20.9
order_Rhodospirillales	rs11630875	T	C	0.095	0.311	15	0.020	3.70E-06	8595	21.9
order_Rhodospirillales	rs13336560	C	T	-0.070	0.336	16	0.016	9.75E-06	8112	19.6
order_Rhodospirillales	rs1549633	A	C	0.100	0.185	5	0.022	3.88E-06	8112	21.2
order_Rhodospirillales	rs3730086	A	G	0.080	0.210	5	0.018	7.98E-06	8602	20.1
order_Rhodospirillales	rs3754624	C	T	0.094	0.215	2	0.020	2.68E-06	8598	22.5
order_Rhodospirillales	rs4278423	T	C	0.105	0.191	10	0.023	3.98E-06	8603	20.3
order_Rhodospirillales	rs4822789	G	C	0.073	0.448	22	0.016	7.33E-06	8594	20.8
order_Rhodospirillales	rs55876211	C	T	-0.087	0.192	3	0.020	7.87E-06	8596	19.6
order_Rhodospirillales	rs61933850	G	A	0.165	0.030	12	0.036	7.00E-06	6846	20.9
order_Rhodospirillales	rs7001029	C	T	0.121	0.110	8	0.026	2.83E-06	8063	21.4
order_Rhodospirillales	rs76784716	A	G	0.136	0.047	2	0.028	1.31E-06	8600	22.9
order_Rhodospirillales	rs77304857	C	A	-0.100	0.125	4	0.022	6.02E-06	8602	20.2
order_Rhodospirillales	rs9813022	A	G	-0.083	0.494	3	0.016	3.07E-07	8600	26.1
family_Rhodospirillaceae	rs1035406	G	A	-0.114	0.191	5	0.025	5.84E-06	8483	20.5
family_Rhodospirillaceae	rs11591293	G	T	0.074	0.163	10	0.016	2.67E-06	8500	21.9
family_Rhodospirillaceae	rs12977163	G	C	0.073	0.226	19	0.016	7.63E-06	8044	20.0
family_Rhodospirillaceae	rs13336560	C	T	-0.070	0.336	16	0.016	9.17E-06	8010	19.7
family_Rhodospirillaceae	rs1549633	A	C	0.100	0.185	5	0.022	4.70E-06	8010	20.9
family_Rhodospirillaceae	rs1923415	A	G	-0.100	0.136	6	0.023	9.64E-06	8501	19.3
family_Rhodospirillaceae	rs3754624	C	T	0.097	0.215	2	0.020	1.71E-06	8496	23.6
family_Rhodospirillaceae	rs4278423	T	C	0.108	0.191	10	0.024	3.12E-06	8501	20.8
family_Rhodospirillaceae	rs4822789	G	C	0.073	0.448	22	0.016	7.01E-06	8492	20.9
family_Rhodospirillaceae	rs55876211	C	T	-0.091	0.192	3	0.020	2.87E-06	8494	21.6
family_Rhodospirillaceae	rs61933850	G	A	0.165	0.030	12	0.036	7.23E-06	6830	20.9
family_Rhodospirillaceae	rs6679026	T	C	0.112	0.106	1	0.025	9.95E-06	8497	19.9
family_Rhodospirillaceae	rs7001029	C	T	0.117	0.110	8	0.026	5.35E-06	8044	20.2
family_Rhodospirillaceae	rs72714493	A	G	0.082	0.174	1	0.018	7.35E-06	8497	20.4
family_Rhodospirillaceae	rs74354280	C	T	-0.091	0.152	4	0.020	6.67E-06	8501	19.8
family_Rhodospirillaceae	rs76784716	A	G	0.136	0.047	2	0.029	1.49E-06	8498	22.7
family_Rhodospirillaceae	rs9813022	A	G	-0.084	0.494	3	0.016	2.53E-07	8498	26.5
family_Rhodospirillaceae	rs10093275	C	T	0.053	0.277	8	0.012	5.35E-06	18335	21.0
family_Rhodospirillaceae	rs10166469	T	C	-0.053	0.239	2	0.012	8.52E-06	18314	19.7
family_Rhodospirillaceae	rs1158100	A	G	-0.049	0.193	8	0.011	8.61E-06	18333	19.9
family_Rhodospirillaceae	rs1612733	T	C	0.109	0.037	1	0.024	4.22E-06	15016	20.9
family_Rhodospirillaceae	rs17376049	T	C	0.085	0.077	1	0.017	7.30E-07	18333	24.3
family_Rhodospirillaceae	rs2113833	T	C	0.169	0.196	2	0.036	1.14E-06	4140	22.7
family_Rhodospirillaceae	rs2426816	T	A	-0.048	0.437	20	0.011	6.04E-06	17774	20.5
family_Rhodospirillaceae	rs3009418	C	A	0.093	0.070	1	0.021	8.69E-06	16407	19.5
family_Rhodospirillaceae	rs499272	G	C	0.071	0.283	11	0.016	9.29E-06	17854	19.4
family_Rhodospirillaceae	rs55793120	T	C	0.138	0.016	12	0.027	1.44E-07	12405	27.0
family_Rhodospirillaceae	rs56199908	T	C	-0.199	0.077	9	0.041	1.66E-06	5618	23.6
family_Rhodospirillaceae	rs76724913	T	G	0.090	0.105	1	0.020	9.60E-06	17516	19.7
genus_Allisonella	rs35778461	C	T	0.147	0.302	9	0.030	1.21E-06	3212	24.4
genus_Allisonella	rs594561	C	T	0.112	0.472	11	0.025	9.41E-06	3212	19.9
genus_Allisonella	rs602075	A	G	0.169	0.124	9	0.030	3.57E-08	3212	32.4
genus_Allisonella	rs6742198	G	A	0.149	0.100	2	0.032	3.35E-06	3182	22.2
genus_Allisonella	rs685403	G	C	-0.175	0.185	18	0.040	4.88E-06	3212	18.8

order_Rhodospirillales	rs11591293	G	T	0.072	0.163	10	0.016	4.69E-06	8602	20.9
order_Rhodospirillales	rs11630875	T	C	0.095	0.311	15	0.020	3.70E-06	8595	21.9
order_Rhodospirillales	rs13336560	C	T	-0.070	0.336	16	0.016	9.75E-06	8112	19.6
order_Rhodospirillales	rs1549633	A	C	0.100	0.185	5	0.022	3.88E-06	8112	21.2
order_Rhodospirillales	rs3730086	A	G	0.080	0.210	5	0.018	7.98E-06	8602	20.1
order_Rhodospirillales	rs3754624	C	T	0.094	0.215	2	0.020	2.68E-06	8598	22.5
order_Rhodospirillales	rs4278423	T	C	0.105	0.191	10	0.023	3.98E-06	8603	20.3
order_Rhodospirillales	rs4822789	G	C	0.073	0.448	22	0.016	7.33E-06	8594	20.8
order_Rhodospirillales	rs55876211	C	T	-0.087	0.192	3	0.020	7.87E-06	8596	19.6
order_Rhodospirillales	rs61933850	G	A	0.165	0.030	12	0.036	7.00E-06	6846	20.9
order_Rhodospirillales	rs7001029	C	T	0.121	0.110	8	0.026	2.83E-06	8063	21.4
order_Rhodospirillales	rs76784716	A	G	0.136	0.047	2	0.028	1.31E-06	8600	22.9
order_Rhodospirillales	rs77304857	C	A	-0.100	0.125	4	0.022	6.02E-06	8602	20.2
order_Rhodospirillales	rs9813022	A	G	-0.083	0.494	3	0.016	3.07E-07	8600	26.1
family_Rhodospirillaceae	rs1035406	G	A	-0.114	0.191	5	0.025	5.84E-06	8483	20.5
family_Rhodospirillaceae	rs11591293	G	T	0.074	0.163	10	0.016	2.67E-06	8500	21.9
family_Rhodospirillaceae	rs12977163	G	C	0.073	0.226	19	0.016	7.63E-06	8044	20.0
family_Rhodospirillaceae	rs13336560	C	T	-0.070	0.336	16	0.016	9.17E-06	8010	19.7
family_Rhodospirillaceae	rs1549633	A	C	0.100	0.185	5	0.022	4.70E-06	8010	20.9
family_Rhodospirillaceae	rs1923415	A	G	-0.100	0.136	6	0.023	9.64E-06	8501	19.3
family_Rhodospirillaceae	rs3754624	C	T	0.097	0.215	2	0.020	1.71E-06	8496	23.6
family_Rhodospirillaceae	rs4278423	T	C	0.108	0.191	10	0.024	3.12E-06	8501	20.8
family_Rhodospirillaceae	rs4822789	G	C	0.073	0.448	22	0.016	7.01E-06	8492	20.9
family_Rhodospirillaceae	rs55876211	C	T	-0.091	0.192	3	0.020	2.87E-06	8494	21.6
family_Rhodospirillaceae	rs61933850	G	A	0.165	0.030	12	0.036	7.23E-06	6830	20.9
family_Rhodospirillaceae	rs6679026	T	C	0.112	0.106	1	0.025	9.95E-06	8497	19.9
family_Rhodospirillaceae	rs7001029	C	T	0.117	0.110	8	0.026	5.35E-06	8044	20.2
family_Rhodospirillaceae	rs72714493	A	G	0.082	0.174	1	0.018	7.35E-06	8497	20.4
family_Rhodospirillaceae	rs74354280	C	T	-0.091	0.152	4	0.020	6.67E-06	8501	19.8
family_Rhodospirillaceae	rs76784716	A	G	0.136	0.047	2	0.029	1.49E-06	8498	22.7
family_Rhodospirillaceae	rs9813022	A	G	-0.084	0.494	3	0.016	2.53E-07	8498	26.5
family_Rhodospirillaceae	rs10093275	C	T	0.053	0.277	8	0.012	5.35E-06	18335	21.0
family_Rhodospirillaceae	rs10166469	T	C	-0.053	0.239	2	0.012	8.52E-06	18314	19.7
family_Rhodospirillaceae	rs1158100	A	G	-0.049	0.193	8	0.011	8.61E-06	18333	19.9
family_Rhodospirillaceae	rs1612733	T	C	0.109	0.037	1	0.024	4.22E-06	15016	20.9
family_Rhodospirillaceae	rs17376049	T	C	0.085	0.077	1	0.017	7.30E-07	18333	24.3
family_Rhodospirillaceae	rs2113833	T	C	0.169	0.196	2	0.036	1.14E-06	4140	22.7
family_Rhodospirillaceae	rs2426816	T	A	-0.048	0.437	20	0.011	6.04E-06	17774	20.5
family_Rhodospirillaceae	rs3009418	C	A	0.093	0.070	1	0.021	8.69E-06	16407	19.5
family_Rhodospirillaceae	rs499272	G	C	0.071	0.283	11	0.016	9.29E-06	17854	19.4
family_Rhodospirillaceae	rs55793120	T	C	0.138	0.016	12	0.027	1.44E-07	12405	27.0
family_Rhodospirillaceae	rs56199908	T	C	-0.199	0.077	9	0.041	1.66E-06	5618	23.6
family_Rhodospirillaceae	rs76724913	T	G	0.090	0.105	1	0.020	9.60E-06	17516	19.7
genus_Allisonella	rs35778461	C	T	0.147	0.302	9	0.030	1.21E-06	3212	24.4
genus_Allisonella	rs594561	C	T	0.112	0.472	11	0.025	9.41E-06	3212	19.9
genus_Allisonella	rs602075	A	G	0.169	0.124	9	0.030	3.57E-08	3212	32.4
genus_Allisonella	rs6742198	G	A	0.149	0.100	2	0.032	3.35E-06	3182	22.2
genus_Allisonella	rs685403	G	C	-0.175	0.185	18	0.040	4.88E-06	3212	18.8
genus_Allisonella	rs76904847	G	A	0.149	0.103	7	0.033	6.09E-06	3182	19.7
genus_Allisonella	rs7898615	T	G	0.168	0.186	10	0.037	8.87E-06	3212	20.2
genus_Coprococcus_2	rs10070053	A	G	0.059	0.227	5	0.014	7.65E-06	11473	19.3
genus_Coprococcus_3	rs10121347	C	G	0.093	0.177	9	0.022	8.31E-06	11457	17.8
genus_Coprococcus_4	rs12634070	T	C	0.074	0.133	3	0.016	9.95E-06	11457	19.9
genus_Coprococcus_5	rs1958519	T	A	0.067	0.270	14	0.014	1.58E-06	11009	23.1
genus_Coprococcus_6	rs2482516	C	T	0.075	0.181	9	0.016	4.72E-06	11483	21.0
genus_Coprococcus_7	rs35890118	A	G	-0.067	0.370	10	0.015	8.26E-06	11478	20.3
genus_Coprococcus_8	rs61823518	A	C	-0.096	0.078	1	0.022	6.68E-06	10980	19.6
genus_Coprococcus_9	rs6677933	C	T	-0.080	0.198	1	0.016	1.19E-06	11483	24.0
genus_Coprococcus_10	rs6894272	T	C	-0.113	0.063	5	0.025	9.53E-06	11001	20.1
genus_Coprococcus_11	rs72680320	T	C	-0.065	0.179	4	0.014	2.27E-06	11009	21.8
genus_Coprococcus_12	rs9426473	A	G	0.073	0.161	1	0.016	6.31E-06	11486	20.2
genus_Holdemanella	rs12415649	G	C	0.084	0.097	10	0.019	7.88E-06	7662	19.4
genus_Holdemanella	rs12513188	G	A	0.090	0.263	4	0.020	4.65E-06	7700	21.4
genus_Holdemanella	rs17586763	T	C	-0.227	0.022	13	0.051	7.72E-06	3817	19.9
genus_Holdemanella	rs1830029	C	G	-0.095	0.187	7	0.021	5.35E-06	7456	20.5
genus_Holdemanella	rs1926302	G	A	-0.108	0.358	1	0.023	7.50E-06	7702	21.8
genus_Holdemanella	rs34187114	C	A	-0.105	0.107	8	0.023	5.13E-06	7704	21.4
genus_Holdemanella	rs35228298	G	A	0.093	0.154	3	0.020	7.30E-06	7456	21.2
genus_Holdemanella	rs4541991	T	C	-0.093	0.162	9	0.019	2.10E-06	7657	22.7
genus_Holdemanella	rs607782	T	C	-0.085	0.309	6	0.017	7.19E-07	7456	24.5
genus_Holdemanella	rs62113381	T	C	-0.105	0.078	19	0.023	5.54E-06	7310	20.7
genus_Holdemanella	rs73011279	T	C	-0.096	0.178	19	0.020	1.36E-06	7456	23.3
genus_Holdemanella	rs761624	C	G	0.096	0.363	6	0.018	1.38E-07	7706	28.6
genus_Holdemanella	rs8113760	G	A	0.079	0.443	19	0.017	4.62E-06	7456	20.8
genus_Prevotella9	rs10512344	C	G	0.247	0.088	9	0.054	3.19E-06	3063	20.7
genus_Prevotella10	rs111509883	T	C	0.171	0.045	19	0.035	1.24E-06	6960	24.2
genus_Prevotella11	rs11199734	A	T	0.077	0.185	10	0.017	7.00E-06	10263	20.7
genus_Prevotella12	rs117271932	A	G	0.208	0.024	22	0.044	2.82E-06	4878	22.3
genus_Prevotella13	rs12648235	T	C	0.079	0.311	4	0.018	7.39E-06	10271	19.6
genus_Prevotella14	rs1304512	G	A	0.076	0.349	5	0.017	5.29E-06	9790	21.1
genus_Prevotella15	rs16966465	G	C	0.074	0.217	15	0.017	9.33E-06	10261	20.2
genus_Prevotella16	rs2104588	T	C	0.106	0.166	10	0.024	8.13E-06	9268	19.7
genus_Prevotella17	rs2495052	A	G	0.084	0.156	1	0.019	8.97E-06	9790	19.8
genus_Prevotella18	rs2683313	A	G	-0.072	0.235	8	0.015	1.69E-06	10260	22.8
genus_Prevotella19	rs4821647	G	C	0.064	0.328	22	0.014	9.95E-06	10179	19.6
genus_Prevotella20	rs4968431	G	T	0.064	0.499	17	0.014	8.58E-06	10271	19.7

family_Rhodospirillaceae	rs3009418	C	A	0.093	0.070	1	0.021	8.69E-06	16407	19.5
family_Rhodospirillaceae	rs499272	G	C	0.071	0.283	11	0.016	9.29E-06	17854	19.4
family_Rhodospirillaceae	rs55793120	T	C	0.138	0.016	12	0.027	1.44E-07	12405	27.0
family_Rhodospirillaceae	rs56199908	T	C	-0.199	0.077	9	0.041	1.66E-06	5618	23.6
family_Rhodospirillaceae	rs76724913	T	G	0.090	0.105	1	0.020	9.60E-06	17516	19.7
genus_Allisonella	rs35778461	C	T	0.147	0.302	9	0.030	1.21E-06	3212	24.4
genus_Allisonella	rs594561	C	T	0.112	0.472	11	0.025	9.41E-06	3212	19.9
genus_Allisonella	rs602075	A	G	0.169	0.124	9	0.030	3.57E-08	3212	32.4
genus_Allisonella	rs6742198	G	A	0.149	0.100	2	0.032	3.35E-06	3182	22.2
genus_Allisonella	rs685403	G	C	-0.175	0.185	18	0.040	4.88E-06	3212	18.8
genus_Allisonella	rs76904847	G	A	0.149	0.103	7	0.033	6.09E-06	3182	19.7
genus_Allisonella	rs7898615	T	G	0.168	0.186	10	0.037	8.87E-06	3212	20.2
genus_Coprococcus_2	rs10070053	A	G	0.059	0.227	5	0.014	7.65E-06	11473	19.3
genus_Coprococcus_3	rs10121347	C	G	0.093	0.177	9	0.022	8.31E-06	11457	17.8
genus_Coprococcus_4	rs12634070	T	C	0.074	0.133	3	0.016	9.95E-06	11457	19.9
genus_Coprococcus_5	rs1958519	T	A	0.067	0.270	14	0.014	1.58E-06	11009	23.1
genus_Coprococcus_6	rs2482516	C	T	0.075	0.181	9	0.016	4.72E-06	11483	21.0
genus_Coprococcus_7	rs35890118	A	G	-0.067	0.370	10	0.015	8.26E-06	11478	20.3
genus_Coprococcus_8	rs61823518	A	C	-0.096	0.078	1	0.022	6.68E-06	10980	19.6
genus_Coprococcus_9	rs6677933	C	T	-0.080	0.198	1	0.016	1.19E-06	11483	24.0
genus_Coprococcus_10	rs6894272	T	C	-0.113	0.063	5	0.025	9.53E-06	11001	20.1
genus_Coprococcus_11	rs72680320	T	C	-0.065	0.179	4	0.014	2.27E-06	11009	21.8
genus_Coprococcus_12	rs9426473	A	G	0.073	0.161	1	0.016	6.31E-06	11486	20.2
genus_Holdemanella	rs12415649	G	C	0.084	0.097	10	0.019	7.88E-06	7662	19.4
genus_Holdemanella	rs12513188	G	A	0.090	0.263	4	0.020	4.65E-06	7700	21.4
genus_Holdemanella	rs17586763	T	C	-0.227	0.022	13	0.051	7.72E-06	3817	19.9
genus_Holdemanella	rs1830029	C	G	-0.095	0.187	7	0.021	5.35E-06	7456	20.5
genus_Holdemanella	rs1926302	G	A	-0.108	0.358	1	0.023	7.50E-06	7702	21.8
genus_Holdemanella	rs34187114	C	A	-0.105	0.107	8	0.023	5.13E-06	7704	21.4
genus_Holdemanella	rs35228298	G	A	0.093	0.154	3	0.020	7.30E-06	7456	21.2
genus_Holdemanella	rs4541991	T	C	-0.093	0.162	9	0.019	2.10E-06	7657	22.7
genus_Holdemanella	rs607782	T	C	-0.085	0.309	6	0.017	7.19E-07	7456	24.5
genus_Holdemanella	rs62113381	T	C	-0.105	0.078	19	0.023	5.54E-06	7310	20.7
genus_Holdemanella	rs73011279	T	C	-0.096	0.178	19	0.020	1.36E-06	7456	23.3
genus_Holdemanella	rs761624	C	G	0.096	0.363	6	0.018	1.38E-07	7706	28.6
genus_Holdemanella	rs8113760	G	A	0.079	0.443	19	0.017	4.62E-06	7456	20.8
genus_Prevotella9	rs10512344	C	G	0.247	0.088	9	0.054	3.19E-06	3063	20.7
genus_Prevotella10	rs111509883	T	C	0.171	0.045	19	0.035	1.24E-06	6960	24.2
genus_Prevotella11	rs11199734	A	T	0.077	0.185	10	0.017	7.00E-06	10263	20.7
genus_Prevotella12	rs117271932	A	G	0.208	0.024	22	0.044	2.82E-06	4878	22.3
genus_Prevotella13	rs12648235	T	C	0.079	0.311	4	0.018	7.39E-06	10271	19.6
genus_Prevotella14	rs1304512	G	A	0.076	0.349	5	0.017	5.29E-06	9790	21.1
genus_Prevotella15	rs16966465	G	C	0.074	0.217	15	0.017	9.33E-06	10261	20.2
genus_Prevotella16	rs2104588	T	C	0.106	0.166	10	0.024	8.13E-06	9268	19.7
genus_Prevotella17	rs2495052	A	G	0.084	0.156	1	0.019	8.97E-06	9790	19.8
genus_Prevotella18	rs2683313	A	G	-0.072	0.235	8	0.015	1.69E-06	10260	22.8
genus_Prevotella19	rs4821647	G	C	0.064	0.328	22	0.014	9.95E-06	10179	19.6
genus_Prevotella20	rs4968431	G	T	0.064	0.499	17	0.014	8.58E-06	10271	19.7
genus_Prevotella21	rs7232121	G	C	0.067	0.487	18	0.014	3.76E-06	9790	21.7
genus_Prevotella22	rs7237249	C	T	-0.082	0.209	18	0.018	8.93E-06	10271	20.5
genus_Prevotella23	rs72815774	T	C	-0.176	0.037	2	0.039	8.78E-06	5728	20.1
genus_Prevotella24	rs746764	T	C	-0.092	0.079	20	0.019	2.04E-06	9385	22.5
genus_Prevotella25	rs9428102	A	G	-0.078	0.150	1	0.018	4.62E-06	10264	19.6
genus_Prevotella26	rs9613013	G	A	0.092	0.246	22	0.020	6.10E-06	9790	20.5
genus_Ruminococcaceae_UCG005	rs10873449	T	C	0.065	0.279	14	0.014	4.11E-06	16098	20.7
genus_Ruminococcaceae_UCG006	rs10937802	G	A	0.076	0.317	4	0.017	8.17E-06	15821	20.2
genus_Ruminococcaceae_UCG007	rs10950694	T	C	0.058	0.384	7	0.011	4.30E-07	15821	25.6
genus_Ruminococcaceae_UCG008	rs114279581	A	G	-0.147	0.058	2	0.032	3.22E-06	8465	21.5
genus_Ruminococcaceae_UCG009	rs12458218	T	C	0.068	0.199	18	0.014	2.41E-06	16099	22.0
genus_Ruminococcaceae_UCG010	rs2893871	G	A	-0.074	0.216	10	0.016	3.54E-06	16103	22.4
genus_Ruminococcaceae_UCG011	rs34781347	G	A	0.189	0.035	20	0.039	6.05E-07	6061	23.8
genus_Ruminococcaceae_UCG012	rs35166120	C	G	-0.069	0.265	8	0.015	3.75E-06	16092	22.0
genus_Ruminococcaceae_UCG013	rs394449	A	T	0.069	0.164	17	0.015	2.60E-06	15728	21.7
genus_Ruminococcaceae_UCG014	rs55793120	T	C	0.122	0.016	12	0.028	7.37E-06	11224	18.9
genus_Ruminococcaceae_UCG015	rs60081663	C	G	0.158	0.026	5	0.032	9.28E-07	9342	24.5
genus_Ruminococcaceae_UCG016	rs72776570	C	A	0.087	0.042	10	0.020	5.36E-06	15376	19.5
genus_Ruminococcaceae_UCG017	rs7449320	C	A	0.060	0.295	5	0.013	4.81E-06	16095	21.0
genus_Ruminococcaceae_UCG018	rs7555878	A	G	0.059	0.442	1	0.013	2.81E-06	16100	21.9
genus_Ruminococcaceae_UCG019	rs7586445	G	A	0.078	0.069	2	0.018	8.81E-06	15099	19.7
genus_Ruminococcaceae_UCG020	rs898577	T	C	-0.123	0.142	15	0.029	7.46E-06	9991	18.4
genus_Ruminococcaceae_UCG014	rs10495392	C	T	-0.082	0.111	1	0.019	9.96E-06	13902	19.4
genus_Ruminococcaceae_UCG015	rs10791168	A	G	-0.066	0.318	11	0.015	9.76E-06	14403	19.6
genus_Ruminococcaceae_UCG016	rs10941294	C	T	-0.122	0.081	5	0.026	2.40E-06	12615	22.0
genus_Ruminococcaceae_UCG017	rs115777838	T	C	-0.188	0.022	5	0.039	4.62E-07	5674	23.7
genus_Ruminococcaceae_UCG018	rs12638134	T	G	0.058	0.378	3	0.012	1.21E-06	14403	23.7
genus_Ruminococcaceae_UCG019	rs17296933	C	G	-0.083	0.054	19	0.019	7.34E-06	13177	20.1
genus_Ruminococcaceae_UCG020	rs34402072	C	T	-0.069	0.124	8	0.016	9.80E-06	13863	19.4
genus_Ruminococcaceae_UCG021	rs439810	G	C	-0.058	0.315	2	0.013	7.04E-06	14403	20.8
genus_Ruminococcaceae_UCG022	rs56105232	G	A	0.139	0.127	9	0.030	2.91E-06	9190	21.7
genus_Ruminococcaceae_UCG023	rs61898819	A	T	0.061	0.151	11	0.014	9.92E-06	14325	19.2
genus_Ruminococcaceae_UCG024	rs62478832	T	A	-0.058	0.175	7	0.013	6.04E-06	14331	20.3
genus_Ruminococcaceae_UCG025	rs73186226	G	A	-0.099	0.044	3	0.022	6.72E-06	13760	21.0
genus_Ruminococcaceae_UCG026	rs74060145	C	G	-0.116	0.077	1	0.025	8.71E-06	13011	20.7
genus_Ruminococcaceae_UCG027	rs79640386	T	A	-0.111	0.094	18	0.025	8.74E-06	13692	20.0
genus_Ruminococcaceae_UCG028	rs995642	C	T	0.060	0.471	2	0.013	1.90E-06	14402	22.6
genus_Ruminococcus_gnavus_group	rs11597105	A	G	0.115	0.078	10	0.025	6.95E-06	6328	20.9

genus_Prevotella10	rs111509883	T	C	0.171	0.045	19	0.035	1.24E-06	6960	24.2
genus_Prevotella11	rs11199734	A	T	0.077	0.185	10	0.017	7.00E-06	10263	20.7
genus_Prevotella12	rs117271932	A	G	0.208	0.024	22	0.044	2.82E-06	4878	22.3
genus_Prevotella13	rs12648235	T	C	0.079	0.311	4	0.018	7.39E-06	10271	19.6
genus_Prevotella14	rs1304512	G	A	0.076	0.349	5	0.017	5.29E-06	9790	21.1
genus_Prevotella15	rs16966465	G	C	0.074	0.217	15	0.017	9.33E-06	10261	20.2
genus_Prevotella16	rs2104588	T	C	0.106	0.166	10	0.024	8.13E-06	9268	19.7
genus_Prevotella17	rs2495052	A	G	0.084	0.156	1	0.019	8.97E-06	9790	19.8
genus_Prevotella18	rs2683313	A	G	-0.072	0.235	8	0.015	1.69E-06	10260	22.8
genus_Prevotella19	rs4821647	G	C	0.064	0.328	22	0.014	9.95E-06	10179	19.6
genus_Prevotella20	rs4968431	G	T	0.064	0.499	17	0.014	8.58E-06	10271	19.7
genus_Prevotella21	rs7232121	G	C	0.067	0.487	18	0.014	3.76E-06	9790	21.7
genus_Prevotella22	rs7237249	C	T	-0.082	0.209	18	0.018	8.93E-06	10271	20.5
genus_Prevotella23	rs72815774	T	C	-0.176	0.037	2	0.039	8.78E-06	5728	20.1
genus_Prevotella24	rs746764	T	C	-0.092	0.079	20	0.019	2.04E-06	9385	22.5
genus_Prevotella25	rs9428102	A	G	-0.078	0.150	1	0.018	4.62E-06	10264	19.6
genus_Prevotella26	rs9613013	G	A	0.092	0.246	22	0.020	6.10E-06	9790	20.5
genus_Ruminococcaceae_UCG005	rs10873449	T	C	0.065	0.279	14	0.014	4.11E-06	16098	20.7
genus_Ruminococcaceae_UCG006	rs10937802	G	A	0.076	0.317	4	0.017	8.17E-06	15821	20.2
genus_Ruminococcaceae_UCG007	rs10950694	T	C	0.058	0.384	7	0.011	4.30E-07	15821	25.6
genus_Ruminococcaceae_UCG008	rs114279581	A	G	-0.147	0.058	2	0.032	3.22E-06	8465	21.5
genus_Ruminococcaceae_UCG009	rs12458218	T	C	0.068	0.199	18	0.014	2.41E-06	16099	22.0
genus_Ruminococcaceae_UCG010	rs2893871	G	A	-0.074	0.216	10	0.016	3.54E-06	16103	22.4
genus_Ruminococcaceae_UCG011	rs34781347	G	A	0.189	0.035	20	0.039	6.05E-07	6061	23.8
genus_Ruminococcaceae_UCG012	rs35166120	C	G	-0.069	0.265	8	0.015	3.75E-06	16092	22.0
genus_Ruminococcaceae_UCG013	rs394449	A	T	0.069	0.164	17	0.015	2.60E-06	15728	21.7
genus_Ruminococcaceae_UCG014	rs55793120	T	C	0.122	0.016	12	0.028	7.37E-06	11224	18.9
genus_Ruminococcaceae_UCG015	rs60081663	C	G	0.158	0.026	5	0.032	9.28E-07	9342	24.5
genus_Ruminococcaceae_UCG016	rs72776570	C	A	0.087	0.042	10	0.020	5.36E-06	15376	19.5
genus_Ruminococcaceae_UCG017	rs7449320	C	A	0.060	0.295	5	0.013	4.81E-06	16095	21.0
genus_Ruminococcaceae_UCG018	rs7555878	A	G	0.059	0.442	1	0.013	2.81E-06	16100	21.9
genus_Ruminococcaceae_UCG019	rs7586445	G	A	0.078	0.069	2	0.018	8.81E-06	15099	19.7
genus_Ruminococcaceae_UCG020	rs898577	T	C	-0.123	0.142	15	0.029	7.46E-06	9991	18.4
genus_Ruminococcaceae_UCG014	rs10495392	C	T	-0.082	0.111	1	0.019	9.96E-06	13902	19.4
genus_Ruminococcaceae_UCG015	rs10791168	A	G	-0.066	0.318	11	0.015	9.76E-06	14403	19.6
genus_Ruminococcaceae_UCG016	rs10941294	C	T	-0.122	0.081	5	0.026	2.40E-06	12615	22.0
genus_Ruminococcaceae_UCG017	rs115777838	T	C	-0.188	0.022	5	0.039	4.62E-07	5674	23.7
genus_Ruminococcaceae_UCG018	rs12638134	T	G	0.058	0.378	3	0.012	1.21E-06	14403	23.7
genus_Ruminococcaceae_UCG019	rs17296933	C	G	-0.083	0.054	19	0.019	7.34E-06	13177	20.1
genus_Ruminococcaceae_UCG020	rs34402072	C	T	-0.069	0.124	8	0.016	9.80E-06	13863	19.4
genus_Ruminococcaceae_UCG021	rs439810	G	C	-0.058	0.315	2	0.013	7.04E-06	14403	20.8
genus_Ruminococcaceae_UCG022	rs56105232	G	A	0.139	0.127	9	0.030	2.91E-06	9190	21.7
genus_Ruminococcaceae_UCG023	rs61898819	A	T	0.061	0.151	11	0.014	9.92E-06	14325	19.2
genus_Ruminococcaceae_UCG024	rs62478832	T	A	-0.058	0.175	7	0.013	6.04E-06	14331	20.3
genus_Ruminococcaceae_UCG025	rs73186226	G	A	-0.099	0.044	3	0.022	6.72E-06	13760	21.0
genus_Ruminococcaceae_UCG026	rs74060145	C	G	-0.116	0.077	1	0.025	8.71E-06	13011	20.7
genus_Ruminococcaceae_UCG027	rs79640386	T	A	-0.111	0.094	18	0.025	8.74E-06	13692	20.0
genus_Ruminococcaceae_UCG028	rs995642	C	T	0.060	0.471	2	0.013	1.90E-06	14402	22.6
genus_Ruminococcus_gnavus_group	rs11597105	A	G	0.115	0.078	10	0.025	6.95E-06	6328	20.9
genus_Ruminococcus_gnavus_group	rs11864644	T	C	-0.140	0.241	16	0.032	5.01E-06	6241	19.3
genus_Ruminococcus_gnavus_group	rs12136548	C	T	0.090	0.126	1	0.020	3.10E-06	6401	21.1
genus_Ruminococcus_gnavus_group	rs13163520	G	A	-0.127	0.144	5	0.023	5.61E-08	6326	29.7
genus_Ruminococcus_gnavus_group	rs2909242	C	A	-0.091	0.487	8	0.018	7.41E-07	6401	24.6
genus_Ruminococcus_gnavus_group	rs3124783	A	G	-0.116	0.164	9	0.025	2.67E-06	6401	21.7
genus_Ruminococcus_gnavus_group	rs4388134	C	T	-0.090	0.374	4	0.020	9.12E-06	6399	19.8
genus_Ruminococcus_gnavus_group	rs62167033	T	C	0.185	0.027	2	0.040	3.50E-06	5194	21.9
genus_Ruminococcus_gnavus_group	rs78399089	T	C	0.144	0.065	3	0.033	6.63E-06	5944	19.6
genus_Ruminococcus_gnavus_group	rs934940	A	C	-0.105	0.292	2	0.023	2.74E-06	6309	20.9
genus_Ruminococcus_gnavus_group	rs9872758	T	C	0.085	0.462	3	0.018	1.66E-06	6398	23.1

Table S4.the selected micobiota SNP which taking into analysis

the *Forward* Two-sample-MR with the loose threshold ($P=5\times10^{-6}$,$R^2=0.001$,window=10000)

Exposure	rsid	effect_allele	other_allele	beta	eaf	CHR	se	pval	samplesize	F
phylum_Firmicutes	rs112334273	G	A	0.063	0.136	21	0.013	9.26E-07	18217	24.3
phylum_Firmicutes	rs13421739	C	G	0.094	0.361	2	0.018	1.95E-06	18340	27.6
phylum_Firmicutes	rs2009919	T	C	0.054	0.446	17	0.011	4.95E-07	17859	25.5
phylum_Firmicutes	rs2332027	G	A	-0.048	0.416	4	0.010	4.05E-06	18340	21.2
phylum_Firmicutes	rs3792064	G	A	0.090	0.153	2	0.018	6.75E-07	17812	24.2
phylum_Firmicutes	rs3852931	C	T	-0.048	0.430	20	0.010	4.53E-06	18260	21.1
phylum_Firmicutes	rs7247191	T	C	-0.071	0.217	19	0.016	4.73E-06	18340	20.5
phylum_Firmicutes	rs7975768	C	G	0.051	0.349	12	0.011	2.82E-06	17859	22.0
order_Rhodospirillales	rs1035406	G	A	-0.115	0.191	5	0.025	4.07E-06	8585	21.4
order_Rhodospirillales	rs11591293	G	T	0.072	0.163	10	0.016	4.69E-06	8602	20.9
order_Rhodospirillales	rs11630875	T	C	0.095	0.311	15	0.020	3.70E-06	8595	21.9
order_Rhodospirillales	rs1549633	A	C	0.100	0.185	5	0.022	3.88E-06	8112	21.2
order_Rhodospirillales	rs3754624	C	T	0.094	0.215	2	0.020	2.68E-06	8598	22.5
order_Rhodospirillales	rs4278423	T	C	0.105	0.191	10	0.023	3.98E-06	8603	20.3
order_Rhodospirillales	rs7001029	C	T	0.121	0.110	8	0.026	2.83E-06	8063	21.4
order_Rhodospirillales	rs76784716	A	G	0.136	0.047	2	0.028	1.31E-06	8600	22.9
order_Rhodospirillales	rs9813022	A	G	-0.083	0.494	3	0.016	3.07E-07	8600	26.1
family_Clostridialesvadin_BB60_group	rs118104867	C	T	0.214	0.039	8	0.046	3.44E-06	4095	22.2
family_Clostridialesvadin_BB60_group	rs13409132	A	G	-0.165	0.171	2	0.035	4.37E-06	5913	22.1
family_Clostridialesvadin_BB60_group	rs2191834	G	T	0.075	0.273	2	0.016	2.50E-06	10511	22.0
family_Clostridialesvadin_BB60_group	rs28691777	C	T	0.137	0.101	17	0.027	6.96E-07	10199	26.4
family_Clostridialesvadin_BB60_group	rs55682560	C	T	-0.132	0.018	15	0.026	4.97E-07	9183	25.3

genus_Ruminococcaceae_UCG019	rs17296933	C	G	-0.083	0.054	19	0.019	7.34E-06	13177	20.1
genus_Ruminococcaceae_UCG020	rs34402072	C	T	-0.069	0.124	8	0.016	9.80E-06	13863	19.4
genus_Ruminococcaceae_UCG021	rs439810	G	C	-0.058	0.315	2	0.013	7.04E-06	14403	20.8
genus_Ruminococcaceae_UCG022	rs56105232	G	A	0.139	0.127	9	0.030	2.91E-06	9190	21.7
genus_Ruminococcaceae_UCG023	rs61898819	A	T	0.061	0.151	11	0.014	9.92E-06	14325	19.2
genus_Ruminococcaceae_UCG024	rs62478832	T	A	-0.058	0.175	7	0.013	6.04E-06	14331	20.3
genus_Ruminococcaceae_UCG025	rs73186226	G	A	-0.099	0.044	3	0.022	6.72E-06	13760	21.0
genus_Ruminococcaceae_UCG026	rs74060145	C	G	-0.116	0.077	1	0.025	8.71E-06	13011	20.7
genus_Ruminococcaceae_UCG027	rs79640386	T	A	-0.111	0.094	18	0.025	8.74E-06	13692	20.0
genus_Ruminococcaceae_UCG028	rs995642	C	T	0.060	0.471	2	0.013	1.90E-06	14402	22.6
genus_Ruminococcus_gnavus_group	rs11597105	A	G	0.115	0.078	10	0.025	6.95E-06	6328	20.9
genus_Ruminococcus_gnavus_group	rs11864644	T	C	-0.140	0.241	16	0.032	5.01E-06	6241	19.3
genus_Ruminococcus_gnavus_group	rs12136548	C	T	0.090	0.126	1	0.020	3.10E-06	6401	21.1
genus_Ruminococcus_gnavus_group	rs13163520	G	A	-0.127	0.144	5	0.023	5.61E-08	6326	29.7
genus_Ruminococcus_gnavus_group	rs2909242	C	A	-0.091	0.487	8	0.018	7.41E-07	6401	24.6
genus_Ruminococcus_gnavus_group	rs3124783	A	G	-0.116	0.164	9	0.025	2.67E-06	6401	21.7
genus_Ruminococcus_gnavus_group	rs4388134	C	T	-0.090	0.374	4	0.020	9.12E-06	6399	19.8
genus_Ruminococcus_gnavus_group	rs62167033	T	C	0.185	0.027	2	0.040	3.50E-06	5194	21.9
genus_Ruminococcus_gnavus_group	rs78399089	T	C	0.144	0.065	3	0.033	6.63E-06	5944	19.6
genus_Ruminococcus_gnavus_group	rs934940	A	C	-0.105	0.292	2	0.023	2.74E-06	6309	20.9
genus_Ruminococcus_gnavus_group	rs9872758	T	C	0.085	0.462	3	0.018	1.66E-06	6398	23.1

Table S4.the selected micobiota SNP which taking into analysis

the *Forward* Two-sample-MR with the loose threshold ($P=5\times10^{-6}$,$R^2=0.001$,window=10000)

Exposure	rsid	effect_allele	other_allele	beta	eaf	CHR	se	pval	samplesize	F
phylum_Firmicutes	rs112334273	G	A	0.063	0.136	21	0.013	9.26E-07	18217	24.3
phylum_Firmicutes	rs13421739	C	G	0.094	0.361	2	0.018	1.95E-06	18340	27.6
phylum_Firmicutes	rs2009919	T	C	0.054	0.446	17	0.011	4.95E-07	17859	25.5
phylum_Firmicutes	rs2332027	G	A	-0.048	0.416	4	0.010	4.05E-06	18340	21.2
phylum_Firmicutes	rs3792064	G	A	0.090	0.153	2	0.018	6.75E-07	17812	24.2
phylum_Firmicutes	rs3852931	C	T	-0.048	0.430	20	0.010	4.53E-06	18260	21.1
phylum_Firmicutes	rs7247191	T	C	-0.071	0.217	19	0.016	4.73E-06	18340	20.5
phylum_Firmicutes	rs7975768	C	G	0.051	0.349	12	0.011	2.82E-06	17859	22.0
order_Rhodospirillales	rs1035406	G	A	-0.115	0.191	5	0.025	4.07E-06	8585	21.4
order_Rhodospirillales	rs11591293	G	T	0.072	0.163	10	0.016	4.69E-06	8602	20.9
order_Rhodospirillales	rs11630875	T	C	0.095	0.311	15	0.020	3.70E-06	8595	21.9
order_Rhodospirillales	rs1549633	A	C	0.100	0.185	5	0.022	3.88E-06	8112	21.2
order_Rhodospirillales	rs3754624	C	T	0.094	0.215	2	0.020	2.68E-06	8598	22.5
order_Rhodospirillales	rs4278423	T	C	0.105	0.191	10	0.023	3.98E-06	8603	20.3
order_Rhodospirillales	rs7001029	C	T	0.121	0.110	8	0.026	2.83E-06	8063	21.4
order_Rhodospirillales	rs76784716	A	G	0.136	0.047	2	0.028	1.31E-06	8600	22.9
order_Rhodospirillales	rs9813022	A	G	-0.083	0.494	3	0.016	3.07E-07	8600	26.1
family_Clostridialesvadin_BB60_group	rs118104867	C	T	0.214	0.039	8	0.046	3.44E-06	4095	22.2
family_Clostridialesvadin_BB60_group	rs13409132	A	G	-0.165	0.171	2	0.035	4.37E-06	5913	22.1
family_Clostridialesvadin_BB60_group	rs2191834	G	T	0.075	0.273	2	0.016	2.50E-06	10511	22.0
family_Clostridialesvadin_BB60_group	rs28691777	C	T	0.137	0.101	17	0.027	6.96E-07	10199	26.4
family_Clostridialesvadin_BB60_group	rs55682560	C	T	-0.132	0.018	15	0.026	4.97E-07	9183	25.3
family_Clostridialesvadin_BB60_group	rs66714985	A	C	0.117	0.146	8	0.025	4.85E-06	10512	21.4
family_Clostridialesvadin_BB60_group	rs7226487	A	G	-0.064	0.453	18	0.014	3.58E-06	10505	21.5
family_Clostridialesvadin_BB60_group	rs7538034	T	G	-0.079	0.181	1	0.017	2.37E-06	10420	22.4
family_Clostridialesvadin_BB60_group	rs7725895	A	G	-0.116	0.266	5	0.024	3.94E-06	10512	23.4
family_Clostridialesvadin_BB60_group	rs9979874	G	C	-0.074	0.376	21	0.015	1.05E-06	10506	24.0
family_Rhodospirillaceae	rs11591293	G	T	0.074	0.163	10	0.016	2.67E-06	8500	21.9
family_Rhodospirillaceae	rs1549633	A	C	0.100	0.185	5	0.022	4.70E-06	8010	20.9
family_Rhodospirillaceae	rs3754624	C	T	0.097	0.215	2	0.020	1.71E-06	8496	23.6
family_Rhodospirillaceae	rs4278423	T	C	0.108	0.191	10	0.024	3.12E-06	8501	20.8
family_Rhodospirillaceae	rs55876211	C	T	-0.091	0.192	3	0.020	2.87E-06	8494	21.6
family_Rhodospirillaceae	rs76784716	A	G	0.136	0.047	2	0.029	1.49E-06	8498	22.7
family_Rhodospirillaceae	rs9813022	A	G	-0.084	0.494	3	0.016	2.53E-07	8498	26.5
family_Ruminococcaceae	rs1612733	T	C	0.109	0.037	1	0.024	4.22E-06	15016	20.9
family_Ruminococcaceae	rs17376049	T	C	0.085	0.077	1	0.017	7.30E-07	18333	24.3
family_Ruminococcaceae	rs2113833	T	C	0.169	0.196	2	0.036	1.14E-06	4140	22.7
family_Ruminococcaceae	rs55793120	T	C	0.138	0.016	12	0.027	1.44E-07	12405	27.0
family_Ruminococcaceae	rs56199908	T	C	-0.199	0.077	9	0.041	1.66E-06	5618	23.6
genus_Allisonella	rs1901739	T	G	0.116	0.445	5	0.025	3.59E-06	3212	21.7
genus_Allisonella	rs35778461	C	T	0.147	0.302	9	0.030	1.21E-06	3212	24.4
genus_Allisonella	rs602075	A	G	0.169	0.124	9	0.030	3.57E-08	3212	32.4
genus_Allisonella	rs6742198	G	A	0.149	0.100	2	0.032	3.35E-06	3182	22.2
genus_Allisonella	rs685403	G	C	-0.175	0.185	18	0.040	4.88E-06	3212	18.8
genus_Coprococcus_2	rs1958519	T	A	0.067	0.270	14	0.014	1.58E-06	11009	23.1
genus_Coprococcus_2	rs2482516	C	T	0.075	0.181	9	0.016	4.72E-06	11483	21.0
genus_Coprococcus_2	rs59936925	A	T	0.117	0.136	20	0.023	9.38E-07	10931	25.0
genus_Coprococcus_2	rs6677933	C	T	-0.080	0.198	1	0.016	1.19E-06	11483	24.0
genus_Coprococcus_2	rs72680320	T	C	-0.065	0.179	4	0.014	2.27E-06	11009	21.8
genus_Holdemanella	rs12513188	G	A	0.090	0.263	4	0.020	4.65E-06	7700	21.4
genus_Holdemanella	rs4541991	T	C	-0.093	0.162	9	0.019	2.10E-06	7657	22.7
genus_Holdemanella	rs607782	T	C	-0.085	0.309	6	0.017	7.19E-07	7456	24.5
genus_Holdemanella	rs73011279	T	C	-0.096	0.178	19	0.020	1.36E-06	7456	23.3
genus_Holdemanella	rs761624	C	G	0.096	0.363	6	0.018	1.38E-07	7706	28.6
genus_Holdemanella	rs8113760	G	A	0.079	0.443	19	0.017	4.62E-06	7456	20.8
genus_Prevotella9	rs10512344	C	G	0.247	0.088	9	0.054	3.19E-06	3063	20.7
genus_Prevotella9	rs111509883	T	C	0.171	0.045	19	0.035	1.24E-06	6960	24.2
genus_Prevotella9	rs11685699	C	T	-0.141	0.031	2	0.030	2.03E-06	8342	22.9
genus_Prevotella9	rs117271932	A	G	0.208	0.024	22	0.044	2.82E-06	4878	22.3
genus_Prevotella9	rs2683313	A	G	-0.072	0.235	8	0.015	1.69E-06	10260	22.8

family_Rhodospirillaceae	rs1549633	A	C	0.100	0.185	5	0.022	4.70E-06	8010	20.9
family_Rhodospirillaceae	rs3754624	C	T	0.097	0.215	2	0.020	1.71E-06	8496	23.6
family_Rhodospirillaceae	rs4278423	T	C	0.108	0.191	10	0.024	3.12E-06	8501	20.8
family_Rhodospirillaceae	rs55876211	C	T	-0.091	0.192	3	0.020	2.87E-06	8494	21.6
family_Rhodospirillaceae	rs76784716	A	G	0.136	0.047	2	0.029	1.49E-06	8498	22.7
family_Rhodospirillaceae	rs9813022	A	G	-0.084	0.494	3	0.016	2.53E-07	8498	26.5
family_Ruminococcaceae	rs1612733	T	C	0.109	0.037	1	0.024	4.22E-06	15016	20.9
family_Ruminococcaceae	rs17376049	T	C	0.085	0.077	1	0.017	7.30E-07	18333	24.3
family_Ruminococcaceae	rs2113833	T	C	0.169	0.196	2	0.036	1.14E-06	4140	22.7
family_Ruminococcaceae	rs55793120	T	C	0.138	0.016	12	0.027	1.44E-07	12405	27.0
family_Ruminococcaceae	rs56199908	T	C	-0.199	0.077	9	0.041	1.66E-06	5618	23.6
genus_Allisonella	rs1901739	T	G	0.116	0.445	5	0.025	3.59E-06	3212	21.7
genus_Allisonella	rs35778461	C	T	0.147	0.302	9	0.030	1.21E-06	3212	24.4
genus_Allisonella	rs602075	A	G	0.169	0.124	9	0.030	3.57E-08	3212	32.4
genus_Allisonella	rs6742198	G	A	0.149	0.100	2	0.032	3.35E-06	3182	22.2
genus_Allisonella	rs685403	G	C	-0.175	0.185	18	0.040	4.88E-06	3212	18.8
genus_Coprococcus_2	rs1958519	T	A	0.067	0.270	14	0.014	1.58E-06	11009	23.1
genus_Coprococcus_2	rs2482516	C	T	0.075	0.181	9	0.016	4.72E-06	11483	21.0
genus_Coprococcus_2	rs59936925	A	T	0.117	0.136	20	0.023	9.38E-07	10931	25.0
genus_Coprococcus_2	rs6677933	C	T	-0.080	0.198	1	0.016	1.19E-06	11483	24.0
genus_Coprococcus_2	rs72680320	T	C	-0.065	0.179	4	0.014	2.27E-06	11009	21.8
genus_Holdemanella	rs12513188	G	A	0.090	0.263	4	0.020	4.65E-06	7700	21.4
genus_Holdemanella	rs4541991	T	C	-0.093	0.162	9	0.019	2.10E-06	7657	22.7
genus_Holdemanella	rs607782	T	C	-0.085	0.309	6	0.017	7.19E-07	7456	24.5
genus_Holdemanella	rs73011279	T	C	-0.096	0.178	19	0.020	1.36E-06	7456	23.3
genus_Holdemanella	rs761624	C	G	0.096	0.363	6	0.018	1.38E-07	7706	28.6
genus_Holdemanella	rs8113760	G	A	0.079	0.443	19	0.017	4.62E-06	7456	20.8
genus_Prevotella9	rs10512344	C	G	0.247	0.088	9	0.054	3.19E-06	3063	20.7
genus_Prevotella9	rs111509883	T	C	0.171	0.045	19	0.035	1.24E-06	6960	24.2
genus_Prevotella9	rs11685699	C	T	-0.141	0.031	2	0.030	2.03E-06	8342	22.9
genus_Prevotella9	rs117271932	A	G	0.208	0.024	22	0.044	2.82E-06	4878	22.3
genus_Prevotella9	rs2683313	A	G	-0.072	0.235	8	0.015	1.69E-06	10260	22.8
genus_Prevotella9	rs7232121	G	C	0.067	0.487	18	0.014	3.76E-06	9790	21.7
genus_Prevotella9	rs746764	T	C	-0.092	0.079	20	0.019	2.04E-06	9385	22.5
genus_Prevotella9	rs9428102	A	G	-0.078	0.150	1	0.018	4.62E-06	10264	19.6
genus_Ruminococcaceae_UCG005	rs10873449	T	C	0.065	0.279	14	0.014	4.11E-06	16098	20.7
genus_Ruminococcaceae_UCG005	rs10950694	T	C	0.058	0.384	7	0.011	4.30E-07	15821	25.6
genus_Ruminococcaceae_UCG005	rs114279581	A	G	-0.147	0.058	2	0.032	3.22E-06	8465	21.5
genus_Ruminococcaceae_UCG005	rs12458218	T	C	0.068	0.199	18	0.014	2.41E-06	16099	22.0
genus_Ruminococcaceae_UCG005	rs2893871	G	A	-0.074	0.216	10	0.016	3.54E-06	16103	22.4
genus_Ruminococcaceae_UCG005	rs34781347	G	A	0.189	0.035	20	0.039	6.05E-07	6061	23.8
genus_Ruminococcaceae_UCG005	rs35166120	C	G	-0.069	0.265	8	0.015	3.75E-06	16092	22.0
genus_Ruminococcaceae_UCG005	rs394449	A	T	0.069	0.164	17	0.015	2.60E-06	15728	21.7
genus_Ruminococcaceae_UCG005	rs60081663	C	G	0.158	0.026	5	0.032	9.28E-07	9342	24.5
genus_Ruminococcaceae_UCG005	rs7449320	C	A	0.060	0.295	5	0.013	4.81E-06	16095	21.0
genus_Ruminococcaceae_UCG005	rs7555878	A	G	0.059	0.442	1	0.013	2.81E-06	16100	21.9
genus_Ruminococcaceae_UCG014	rs10941294	C	T	-0.122	0.081	5	0.026	2.40E-06	12615	22.0
genus_Ruminococcaceae_UCG014	rs115777838	T	C	-0.188	0.022	5	0.039	4.62E-07	5674	23.7
genus_Ruminococcaceae_UCG014	rs12638134	T	G	0.058	0.378	3	0.012	1.21E-06	14403	23.7
genus_Ruminococcaceae_UCG014	rs56105232	G	A	0.139	0.127	9	0.030	2.91E-06	9190	21.7
genus_Ruminococcaceae_UCG014	rs995642	C	T	0.060	0.471	2	0.013	1.90E-06	14402	22.6
genus_Ruminococcus_gnavus_group	rs12136548	C	T	0.090	0.126	1	0.020	3.10E-06	6401	21.1
genus_Ruminococcus_gnavus_group	rs13163520	G	A	-0.127	0.144	5	0.023	5.61E-08	6326	29.7
genus_Ruminococcus_gnavus_group	rs2909242	C	A	-0.091	0.487	8	0.018	7.41E-07	6401	24.6
genus_Ruminococcus_gnavus_group	rs3124783	A	G	-0.116	0.164	9	0.025	2.67E-06	6401	21.7
genus_Ruminococcus_gnavus_group	rs62167033	T	C	0.185	0.027	2	0.040	3.50E-06	5194	21.9
genus_Ruminococcus_gnavus_group	rs934940	A	C	-0.105	0.292	2	0.023	2.74E-06	6309	20.9
genus_Ruminococcus_gnavus_group	rs9872758	T	C	0.085	0.462	3	0.018	1.66E-06	6398	23.1

Revese analysis on two different thresholds

Table S5. Excluded pQTLs that were ill-annotated in RegulomeDB database in terms of allele frequency in European descendants

the *reverse* Two-sample-MR with the loose threshold ($P=1\times10^{-5}$,$R^2=0.01$,window=10000)

Exposure	rsid	CHR	POS	effect_allele	other_allele	beta	se	eaf	pval
PD-1	rs372489804	10	23262222	t	ttaa	0.2444	0.0514	0.07297	2.04E-06
PD-1	rs34480730	6	8241682	ca	c	-0.1635	0.0356	0.17793	4.37E-06
PD-1	rs10674947	22	22712021	ccggg	c	0.1636	0.0286	0.27342	1.07E-08
PD-1	rs553317558	22	22712022	g	ga	-0.155	0.0283	0.28101	4.27E-08
PD-1	rs56174980	22	22714875	a	at	0.3751	0.0399	0.10633	5.62E-21
PD-1	rs397779565	22	22715293	a	at	-0.1698	0.0293	0.24054	7.08E-09
PD-1	rs77360830	22	22715601	ct	c	-0.1641	0.0288	0.23217	1.15E-08
PD-1	rs60704833	22	22716011	t	tc	0.1764	0.0308	0.20848	1.05E-08
PD-1	rs11281871	22	22720439	g	gtttctctac	-0.1623	0.0288	0.23247	1.78E-08

PD-1	rs11324703	22	22724135	g	gt	0.3715	0.0401	0.10557	2.09E-20
PD-1	rs738882	22	22727099	t	g	-0.3433	0.0454	0.08121	3.72E-14
PD-1	rs529047042	15	24492759	c	g	-0.1818	0.0379	0.14306	1.62E-06
PD-1	rs200295944	15	24492777	t	g	-0.1825	0.0377	0.14708	1.29E-06
PD-1	rs376272873	15	24492796	t	g	0.1772	0.0377	0.14683	2.51E-06
PD-1	rs200459383	15	24494763	c	g	-0.1741	0.0371	0.14889	2.69E-06
PD-1	rs530862429	16	25642394	g	gtgca	0.3826	0.082	0.024008	3.09E-06
PD-1	rs199553944	16	25667950	t	ta	0.3702	0.0801	0.024152	3.80E-06
PD-1	rs115021813	14	95141475	t	g	0.3983	0.0838	0.029446	2.00E-06
PD-1	rs200972411	22	22719654	a	g	-0.121	0.0268	0.42785	6.46E-06
PD-1	rs76781291	22	31846882	a	c	0.2621	0.0577	0.062617	5.50E-06
PD-1	rs200401952	15	24486050	a	acg	0.1866	0.0415	0.12461	6.76E-06
PD-1	rs199992172	15	24489744	t	c	-0.1493	0.0331	0.17919	6.61E-06
PD-1	rs374293606	15	24492669	t	g	-0.1592	0.0354	0.15988	6.92E-06
PD-1	rs112249730	15	24493108	a	g	0.1444	0.0326	0.1796	9.55E-06
PD-1	rs148483151	15	24494596	c	g	-0.1702	0.0379	0.14589	7.24E-06
PD-1	rs141283926	15	24494725	a	g	-0.1667	0.0371	0.14795	7.24E-06
PD-1	rs140082449	15	24508923	c	g	-0.1476	0.033	0.17926	7.59E-06
PD-1	rs5778700	1	1.71E+08	ca	c	0.1122	0.0252	0.48849	8.32E-06
PD-L1	rs35535800	5	89016534	t	ttg	0.3011	0.0641	0.049734	2.69E-06
PD-L1	rs79698189	6	1.67E+08	a	c	-0.1293	0.0279	0.34171	3.63E-06
PD-L1	rs11397003	8	1.35E+08	t	tc	-0.1195	0.025	0.46216	1.82E-06
PD-L1	rs397693763	9	5460003	g	gtc	0.1568	0.0278	0.27573	1.74E-08
PD-L1	rs186389777	10	426088	a	t	-0.2003	0.0431	0.091366	3.31E-06
PD-L1	rs190748362	10	426100	t	c	-0.2053	0.0433	0.089639	2.14E-06
PD-L1	rs11390566	10	1.12E+08	ct	c	-0.2188	0.0459	0.088625	1.86E-06
PD-L1	rs7144724	14	1.07E+08	a	g	0.1431	0.0307	0.26852	3.16E-06
PD-L1	rs7146933	14	1.07E+08	c	g	-0.145	0.0279	0.26534	2.04E-07
PD-L1	rs35005563	14	1.07E+08	t	g	0.1453	0.0293	0.25671	6.92E-07
PD-L1	rs201179349	14	1.07E+08	t	c	0.1393	0.0293	0.25869	2.00E-06
PD-L1	rs78367943	14	1.07E+08	t	c	-0.1393	0.0293	0.25869	2.00E-06
PD-L1	rs34891277	14	1.07E+08	a	g	0.1393	0.0293	0.25869	2.00E-06
PD-L1	rs113981590	14	1.07E+08	cacccatt	c	0.1489	0.028	0.26556	1.00E-07
PD-L1	rs36036916	14	1.07E+08	a	ag	0.1469	0.0278	0.26732	1.26E-07
PD-L1	rs145456466	14	1.07E+08	g	ggc	-0.1451	0.0302	0.24061	1.51E-06
PD-L1	rs35162884	14	1.07E+08	cat	c	0.1625	0.0269	0.30554	1.58E-09
PD-L1	rs148434864	14	1.07E+08	t	tatta	-0.1617	0.0275	0.28681	3.98E-09
PD-L1	rs200630742	14	1.07E+08	t	c	-0.1568	0.0275	0.28871	1.20E-08
PD-L1	rs142218925	14	1.07E+08	ccaaa	c	0.1548	0.0271	0.29684	1.10E-08
PD-L1	rs34547344	14	1.07E+08	t	tg	-0.1617	0.0267	0.30378	1.38E-09
PD-L1	rs72622042	14	1.07E+08	a	t	0.1608	0.0267	0.30445	1.62E-09
PD-L1	rs11353283	14	1.07E+08	g	gt	0.1358	0.0256	0.49134	1.10E-07
PD-L1	rs12641097	4	20086129	t	g	-0.2634	0.059	0.063465	7.94E-06
PD-L1	rs3043374	9	5440075	a	aacat	0.2314	0.0512	0.068949	6.17E-06

PD-L1	rs199685156	10	426101	a	g	0.2159	0.0474	0.079718	5.25E-06
PD-L1	rs112399670	10	20378078	t	ta	0.2961	0.0669	0.03558	9.55E-06
PD-L1	rs145162348	10	20383344	a	at	-0.3001	0.0676	0.033922	8.91E-06
PD-L1	rs145030604	10	20456034	g	gaa	0.3028	0.0671	0.034967	6.31E-06
PD-L1	rs2257349	14	1.07E+08	t	c	0.1345	0.0304	0.27505	9.55E-06
PD-L1	rs11160977	14	1.07E+08	t	c	-0.1352	0.0301	0.27788	7.24E-06
PD-L1	rs66825724	14	1.07E+08	a	attt	0.121	0.0271	0.31787	7.94E-06
PD-L1	rs34705140	14	1.07E+08	t	tcc	0.1345	0.0298	0.33012	6.46E-06
PD-L1	rs12641097	4	20086129	t	g	-0.2634	0.059	0.063465	7.94E-06
PD-L1	rs3043374	9	5440075	a	aacat	0.2314	0.0512	0.068949	6.17E-06
PD-L1	rs199685156	10	426101	a	g	0.2159	0.0474	0.079718	5.25E-06
PD-L1	rs112399670	10	20378078	t	ta	0.2961	0.0669	0.03558	9.55E-06
PD-L1	rs145162348	10	20383344	a	at	-0.3001	0.0676	0.033922	8.91E-06
PD-L1	rs145030604	10	20456034	g	gaa	0.3028	0.0671	0.034967	6.31E-06
PD-L1	rs2257349	14	1.07E+08	t	c	0.1345	0.0304	0.27505	9.55E-06
PD-L1	rs11160977	14	1.07E+08	t	c	-0.1352	0.0301	0.27788	7.24E-06
PD-L1	rs66825724	14	1.07E+08	a	attt	0.121	0.0271	0.31787	7.94E-06
PD-L1	rs34705140	14	1.07E+08	t	tcc	0.1345	0.0298	0.33012	6.46E-06

the *reverse* Two-sample-MR with the loose threshold ($P=5\times10^{-6}$,$R^2=0.001$,window=10000)

Exposure	rsid	CHR	POS	effect_allele	other_allele	beta	se	eaf	pval
PD-1	rs372489804	10	23262222	t	ttaa	0.2444	0.0514	0.07297	2.04E-06
PD-1	rs34480730	6	8241682	ca	c	-0.1635	0.0356	0.17793	4.37E-06
PD-1	rs10674947	22	22712021	ccggg	c	0.1636	0.0286	0.27342	1.07E-08
PD-1	rs553317558	22	22712022	g	ga	-0.155	0.0283	0.28101	4.27E-08
PD-1	rs56174980	22	22714875	a	at	0.3751	0.0399	0.10633	5.62E-21
PD-1	rs397779565	22	22715293	a	at	-0.1698	0.0293	0.24054	7.08E-09
PD-1	rs77360830	22	22715601	ct	c	-0.1641	0.0288	0.23217	1.15E-08
PD-1	rs60704833	22	22716011	t	tc	0.1764	0.0308	0.20848	1.05E-08
PD-1	rs11281871	22	22720439	g	gtttctctac	-0.1623	0.0288	0.23247	1.78E-08
PD-1	rs11324703	22	22724135	g	gt	0.3715	0.0401	0.10557	2.09E-20
PD-1	rs738882	22	22727099	t	g	-0.3433	0.0454	0.08121	3.72E-14
PD-1	rs529047042	15	24492759	c	g	-0.1818	0.0379	0.14306	1.62E-06
PD-1	rs200295944	15	24492777	t	g	-0.1825	0.0377	0.14708	1.29E-06
PD-1	rs376272873	15	24492796	t	g	0.1772	0.0377	0.14683	2.51E-06
PD-1	rs200459383	15	24494763	c	g	-0.1741	0.0371	0.14889	2.69E-06
PD-1	rs530862429	16	25642394	g	gtgca	0.3826	0.082	0.024008	3.09E-06
PD-1	rs199553944	16	25667950	t	ta	0.3702	0.0801	0.024152	3.80E-06
PD-1	rs115021813	14	95141475	t	g	0.3983	0.0838	0.029446	2.00E-06
PD-L1	rs35535800	5	89016534	t	ttg	0.3011	0.0641	0.049734	2.69E-06
PD-L1	rs79698189	6	1.67E+08	a	c	-0.1293	0.0279	0.34171	3.63E-06
PD-L1	rs11397003	8	1.35E+08	t	tc	-0.1195	0.025	0.46216	1.82E-06
PD-L1	rs397693763	9	5460003	g	gtc	0.1568	0.0278	0.27573	1.74E-08
PD-L1	rs186389777	10	426088	a	t	-0.2003	0.0431	0.091366	3.31E-06
PD-L1	rs190748362	10	426100	t	c	-0.2053	0.0433	0.089639	2.14E-06

PD-L1	rs11390566	10	1.12E+08	ct	c	-0.2188	0.0459	0.088625	1.86E-06
PD-L1	rs7144724	14	1.07E+08	a	g	0.1431	0.0307	0.26852	3.16E-06
PD-L1	rs7146933	14	1.07E+08	c	g	-0.145	0.0279	0.26534	2.04E-07
PD-L1	rs35005563	14	1.07E+08	t	g	0.1453	0.0293	0.25671	6.92E-07
PD-L1	rs201179349	14	1.07E+08	t	c	0.1393	0.0293	0.25869	2.00E-06
PD-L1	rs78367943	14	1.07E+08	t	c	-0.1393	0.0293	0.25869	2.00E-06
PD-L1	rs34891277	14	1.07E+08	a	g	0.1393	0.0293	0.25869	2.00E-06
PD-L1	rs113981590	14	1.07E+08	cacccatt	c	0.1489	0.028	0.26556	1.00E-07
PD-L1	rs36036916	14	1.07E+08	a	ag	0.1469	0.0278	0.26732	1.26E-07
PD-L1	rs145456466	14	1.07E+08	g	ggc	-0.1451	0.0302	0.24061	1.51E-06
PD-L1	rs35162884	14	1.07E+08	cat	c	0.1625	0.0269	0.30554	1.58E-09
PD-L1	rs148434864	14	1.07E+08	t	tatta	-0.1617	0.0275	0.28681	3.98E-09
PD-L1	rs200630742	14	1.07E+08	t	c	-0.1568	0.0275	0.28871	1.20E-08
PD-L1	rs142218925	14	1.07E+08	ccaaa	c	0.1548	0.0271	0.29684	1.10E-08
PD-L1	rs34547344	14	1.07E+08	t	tg	-0.1617	0.0267	0.30378	1.38E-09
PD-L1	rs72622042	14	1.07E+08	a	t	0.1608	0.0267	0.30445	1.62E-09
PD-L1	rs11353283	14	1.07E+08	g	gt	0.1358	0.0256	0.49134	1.10E-07

Table S6. Excluded *tran*-pQLTs that were associated with expression quantitative trait loci (eQTLs) in RegulomeDB database the *reverse* Two-sample-MR with the loose threshold ($P=1\times10^{-5}$,$R^2=0.01$,window=10000)

Exposure	SNP	CHR	POS	effect_allele	other_allele	beta	se	eaf	pval
PD-1	rs2378606	1	2.23E+08	a	g	-0.1448	0.0313	0.18872	3.80E-06
PD-1	rs6683071	1	2.23E+08	a	g	0.1441	0.0314	0.1875	4.37E-06
PD-1	rs12073392	1	2.23E+08	a	c	-0.1455	0.0314	0.18728	3.72E-06
PD-1	rs5030928	10	71176637	a	g	-0.1509	0.0293	0.26207	2.69E-07
PD-1	rs138691176	10	71180887	a	g	0.338	0.0696	0.037564	1.20E-06
PD-1	rs5757973	22	22712467	t	g	-0.3788	0.0399	0.10972	2.00E-21
PD-1	rs3753078	22	22712742	a	g	-0.1654	0.0288	0.23271	9.33E-09
PD-1	rs4821951	22	22712810	c	g	-0.3737	0.0401	0.10629	1.07E-20
PD-1	rs4821952	22	22712831	t	c	0.1665	0.0289	0.23064	8.51E-09
PD-1	rs6001947	22	22712891	a	g	0.1227	0.0246	0.4964	6.31E-07
PD-1	rs1573599	22	22713236	a	g	-0.1658	0.0288	0.23199	8.51E-09
PD-1	rs1015184	22	22713451	t	c	-0.1243	0.0246	0.49689	4.17E-07
PD-1	rs1015183	22	22713585	c	g	-0.1661	0.0288	0.23179	7.94E-09
PD-1	rs1015182	22	22713917	a	g	0.1651	0.0288	0.2318	1.00E-08
PD-1	rs4821954	22	22714084	a	g	0.3757	0.0399	0.10654	5.01E-21
PD-1	rs4821955	22	22714403	t	c	-0.164	0.0288	0.2321	1.23E-08
PD-1	rs5757982	22	22714459	a	g	-0.3756	0.0399	0.10653	4.90E-21
PD-1	rs5757987	22	22714817	c	g	0.1665	0.0288	0.23126	7.41E-09
PD-1	rs5757988	22	22715050	a	g	-0.1641	0.0288	0.23192	1.17E-08
PD-1	rs5750955	22	22715494	t	g	0.165	0.0288	0.23227	9.55E-09
PD-1	rs4413251	22	22715979	a	t	0.1633	0.0288	0.23201	1.41E-08
PD-1	rs2330107	22	22716405	c	g	0.1651	0.0288	0.23188	9.55E-09
PD-1	rs5757999	22	22716568	a	g	0.1641	0.0289	0.23129	1.29E-08
PD-1	rs2877051	22	22716603	a	t	0.164	0.0288	0.23182	1.23E-08

PD-1	rs2213144	22	22716750	a	g	-0.1667	0.0288	0.23219	6.92E-09
PD-1	rs2213150	22	22717444	t	c	0.1643	0.0288	0.23208	1.12E-08
PD-1	rs2330110	22	22717544	a	g	0.166	0.0288	0.23198	7.76E-09
PD-1	rs2078662	22	22717616	a	g	0.3726	0.0399	0.10663	9.77E-21
PD-1	rs5758005	22	22717874	a	t	-0.1649	0.0288	0.23194	1.00E-08
PD-1	rs4821958	22	22718266	a	g	-0.1663	0.0288	0.23188	7.41E-09
PD-1	rs5758009	22	22718635	t	c	-0.378	0.0399	0.10644	2.88E-21
PD-1	rs7288435	22	22718951	a	c	0.1263	0.0248	0.49619	3.39E-07
PD-1	rs5750963	22	22718988	t	c	-0.1689	0.0288	0.23151	4.57E-09
PD-1	rs5750965	22	22719051	t	c	-0.1654	0.0288	0.23218	9.77E-09
PD-1	rs1988718	22	22719296	a	c	-0.1673	0.0289	0.23151	6.76E-09
PD-1	rs5758010	22	22719864	a	t	-0.1654	0.0288	0.23294	9.12E-09
PD-1	rs6001967	22	22719884	c	g	0.3717	0.0398	0.10663	1.07E-20
PD-1	rs5758011	22	22719989	a	g	0.1251	0.0248	0.49616	4.37E-07
PD-1	rs4821960	22	22720055	a	g	-0.1653	0.0288	0.23219	9.55E-09
PD-1	rs5750969	22	22720715	a	g	0.1637	0.0288	0.2321	1.32E-08
PD-1	rs5758013	22	22720928	a	g	0.1621	0.0277	0.25559	5.01E-09
PD-1	rs5758014	22	22720982	a	c	0.1584	0.0279	0.25381	1.35E-08
PD-1	rs5758015	22	22720983	t	g	-0.16	0.0279	0.25423	9.55E-09
PD-1	rs5758016	22	22721022	t	c	0.1617	0.0277	0.25548	5.25E-09
PD-1	rs762467	22	22721878	a	c	0.1639	0.0279	0.25619	4.17E-09
PD-1	rs762468	22	22721975	a	g	-0.1642	0.0279	0.25577	3.80E-09
PD-1	rs6519253	22	22722482	t	c	0.3663	0.04	0.10587	5.37E-20
PD-1	rs4820413	22	22722733	t	c	-0.1645	0.028	0.25803	4.07E-09
PD-1	rs4820414	22	22722770	a	g	-0.1642	0.0281	0.25472	4.90E-09
PD-1	rs995194	22	22722929	a	g	0.1749	0.0289	0.23666	1.35E-09
PD-1	rs7292000	22	22724394	t	c	-0.3681	0.0401	0.10591	4.17E-20
PD-1	rs5758027	22	22725114	a	t	0.3527	0.0409	0.10335	6.31E-18
PD-1	rs3937349	22	22725550	t	c	0.1584	0.0255	0.40274	5.01E-10
PD-1	rs4821969	22	22729881	a	g	0.154	0.0258	0.38535	2.45E-09
PD-1	rs2009976	22	22731960	a	c	-0.1548	0.0262	0.35501	3.31E-09
PD-1	rs5750979	22	22732246	a	g	0.1489	0.0261	0.35712	1.20E-08
PD-1	rs111422459	22	22738468	a	c	-0.411	0.0582	0.053256	1.70E-12
PD-1	rs35806535	1	2.23E+08	t	c	0.1402	0.0311	0.1934	6.76E-06
PD-1	rs75027584	1	2.23E+08	t	g	-0.1382	0.0312	0.19255	9.33E-06
PD-1	rs502096	6	8238578	a	g	-0.1476	0.0331	0.18001	8.13E-06
PD-1	rs73529574	6	1.1E+08	a	g	0.2967	0.0667	0.036814	8.71E-06
PD-1	rs12874732	13	67956074	a	g	0.1346	0.0296	0.2344	5.50E-06
PD-1	rs61959555	13	67972681	a	g	0.1278	0.0285	0.25962	7.41E-06
PD-1	rs35062695	15	33559384	t	g	-0.1336	0.0302	0.21834	9.77E-06
PD-L1	rs822335	9	5448218	t	c	-0.1267	0.0261	0.35209	1.20E-06
PD-L1	rs822337	9	5449154	a	t	0.1262	0.0261	0.349	1.41E-06
PD-L1	rs822338	9	5451557	t	c	0.1603	0.0276	0.27368	6.46E-09
PD-L1	rs822339	9	5453172	a	g	-0.1716	0.0276	0.26524	5.25E-10

PD-L1	rs860290	9	5453198	a	c	-0.1719	0.0276	0.26493	5.01E-10
PD-L1	rs822340	9	5453260	a	g	-0.1717	0.0278	0.26512	6.31E-10
PD-L1	rs822341	9	5453396	t	c	-0.1731	0.0278	0.2653	4.47E-10
PD-L1	rs822342	9	5453973	t	c	-0.1707	0.0277	0.26512	7.59E-10
PD-L1	rs2282055	9	5455732	t	g	0.1628	0.0278	0.26607	4.90E-09
PD-L1	rs17742278	9	5456523	t	c	0.1583	0.0277	0.27614	1.07E-08
PD-L1	rs1411262	9	5459419	t	c	-0.1657	0.028	0.26669	3.39E-09
PD-L1	rs10114060	9	5461729	a	g	-0.1556	0.0279	0.27124	2.57E-08
PD-L1	rs7041009	9	5463243	a	g	-0.1572	0.028	0.26931	2.09E-08
PD-L1	rs11011802	10	20393945	a	c	-0.3054	0.0665	0.035951	4.37E-06
PD-L1	rs11011804	10	20394779	a	g	0.3091	0.0671	0.0353	4.07E-06
PD-L1	rs57927904	11	1.05E+08	t	c	-0.1976	0.0442	0.085353	7.76E-06
PD-L1	rs17102902	11	1.05E+08	t	c	-0.1954	0.0441	0.085694	9.33E-06
PD-L1	rs7952332	11	1.25E+08	t	c	0.1299	0.0293	0.24376	9.12E-06
PD-L1	rs79643961	10	428383	a	g	-0.2132	0.0479	0.071531	8.32E-06
PD-L1	rs3780394	9	5464376	a	c	0.2137	0.0481	0.071728	9.12E-06
PD-L1	rs3780394	9	5464376	a	c	0.2137	0.0481	0.071728	9.12E-06
PD-L1	rs57927904	11	1.05E+08	t	c	-0.1976	0.0442	0.085353	7.76E-06
PD-L1	rs17102902	11	1.05E+08	t	c	-0.1954	0.0441	0.085694	9.33E-06
PD-L1	rs7952332	11	1.25E+08	t	c	0.1299	0.0293	0.24376	9.12E-06

the *reverse* Two-sample-MR with the loose threshold ($P=5\times10^{-6}$,$R^2=0.001$,window=10000)

Exposure	rsid	CHR	POS	effect_allele	other_allele	beta	se	eaf	pval
PD-1	rs2378606	1	2.23E+08	a	g	-0.1448	0.0313	0.18872	3.80E-06
PD-1	rs6683071	1	2.23E+08	a	g	0.1441	0.0314	0.1875	4.37E-06
PD-1	rs12073392	1	2.23E+08	a	c	-0.1455	0.0314	0.18728	3.72E-06
PD-1	rs5030928	10	71176637	a	g	-0.1509	0.0293	0.26207	2.69E-07
PD-1	rs138691176	10	71180887	a	g	0.338	0.0696	0.037564	1.20E-06
PD-1	rs5757973	22	22712467	t	g	-0.3788	0.0399	0.10972	2.00E-21
PD-1	rs3753078	22	22712742	a	g	-0.1654	0.0288	0.23271	9.33E-09
PD-1	rs4821951	22	22712810	c	g	-0.3737	0.0401	0.10629	1.07E-20
PD-1	rs4821952	22	22712831	t	c	0.1665	0.0289	0.23064	8.51E-09
PD-1	rs6001947	22	22712891	a	g	0.1227	0.0246	0.4964	6.31E-07
PD-1	rs1573599	22	22713236	a	g	-0.1658	0.0288	0.23199	8.51E-09
PD-1	rs1015184	22	22713451	t	c	-0.1243	0.0246	0.49689	4.17E-07
PD-1	rs1015183	22	22713585	c	g	-0.1661	0.0288	0.23179	7.94E-09
PD-1	rs1015182	22	22713917	a	g	0.1651	0.0288	0.2318	1.00E-08
PD-1	rs4821954	22	22714084	a	g	0.3757	0.0399	0.10654	5.01E-21
PD-1	rs4821955	22	22714403	t	c	-0.164	0.0288	0.2321	1.23E-08
PD-1	rs5757982	22	22714459	a	g	-0.3756	0.0399	0.10653	4.90E-21
PD-1	rs5757987	22	22714817	c	g	0.1665	0.0288	0.23126	7.41E-09
PD-1	rs5757988	22	22715050	a	g	-0.1641	0.0288	0.23192	1.17E-08
PD-1	rs5750955	22	22715494	t	g	0.165	0.0288	0.23227	9.55E-09
PD-1	rs4413251	22	22715979	a	t	0.1633	0.0288	0.23201	1.41E-08
PD-1	rs2330107	22	22716405	c	g	0.1651	0.0288	0.23188	9.55E-09

PD-1	rs5757999	22	22716568	a	g	0.1641	0.0289	0.23129	1.29E-08
PD-1	rs2877051	22	22716603	a	t	0.164	0.0288	0.23182	1.23E-08
PD-1	rs2213144	22	22716750	a	g	-0.1667	0.0288	0.23219	6.92E-09
PD-1	rs2213150	22	22717444	t	c	0.1643	0.0288	0.23208	1.12E-08
PD-1	rs2330110	22	22717544	a	g	0.166	0.0288	0.23198	7.76E-09
PD-1	rs2078662	22	22717616	a	g	0.3726	0.0399	0.10663	9.77E-21
PD-1	rs5758005	22	22717874	a	t	-0.1649	0.0288	0.23194	1.00E-08
PD-1	rs4821958	22	22718266	a	g	-0.1663	0.0288	0.23188	7.41E-09
PD-1	rs5758009	22	22718635	t	c	-0.378	0.0399	0.10644	2.88E-21
PD-1	rs7288435	22	22718951	a	c	0.1263	0.0248	0.49619	3.39E-07
PD-1	rs5750963	22	22718988	t	c	-0.1689	0.0288	0.23151	4.57E-09
PD-1	rs5750965	22	22719051	t	c	-0.1654	0.0288	0.23218	9.77E-09
PD-1	rs1988718	22	22719296	a	c	-0.1673	0.0289	0.23151	6.76E-09
PD-1	rs5758010	22	22719864	a	t	-0.1654	0.0288	0.23294	9.12E-09
PD-1	rs6001967	22	22719884	c	g	0.3717	0.0398	0.10663	1.07E-20
PD-1	rs5758011	22	22719989	a	g	0.1251	0.0248	0.49616	4.37E-07
PD-1	rs4821960	22	22720055	a	g	-0.1653	0.0288	0.23219	9.55E-09
PD-1	rs5750969	22	22720715	a	g	0.1637	0.0288	0.2321	1.32E-08
PD-1	rs5758013	22	22720928	a	g	0.1621	0.0277	0.25559	5.01E-09
PD-1	rs5758014	22	22720982	a	c	0.1584	0.0279	0.25381	1.35E-08
PD-1	rs5758015	22	22720983	t	g	-0.16	0.0279	0.25423	9.55E-09
PD-1	rs5758016	22	22721022	t	c	0.1617	0.0277	0.25548	5.25E-09
PD-1	rs762467	22	22721878	a	c	0.1639	0.0279	0.25619	4.17E-09
PD-1	rs762468	22	22721975	a	g	-0.1642	0.0279	0.25577	3.80E-09
PD-1	rs6519253	22	22722482	t	c	0.3663	0.04	0.10587	5.37E-20
PD-1	rs4820413	22	22722733	t	c	-0.1645	0.028	0.25803	4.07E-09
PD-1	rs4820414	22	22722770	a	g	-0.1642	0.0281	0.25472	4.90E-09
PD-1	rs995194	22	22722929	a	g	0.1749	0.0289	0.23666	1.35E-09
PD-1	rs7292000	22	22724394	t	c	-0.3681	0.0401	0.10591	4.17E-20
PD-1	rs5758027	22	22725114	a	t	0.3527	0.0409	0.10335	6.31E-18
PD-1	rs3937349	22	22725550	t	c	0.1584	0.0255	0.40274	5.01E-10
PD-1	rs4821969	22	22729881	a	g	0.154	0.0258	0.38535	2.45E-09
PD-1	rs2009976	22	22731960	a	c	-0.1548	0.0262	0.35501	3.31E-09
PD-1	rs5750979	22	22732246	a	g	0.1489	0.0261	0.35712	1.20E-08
PD-1	rs111422459	22	22738468	a	c	-0.411	0.0582	0.053256	1.70E-12
PD-L1	rs822335	9	5448218	t	c	-0.1267	0.0261	0.35209	1.20E-06
PD-L1	rs822337	9	5449154	a	t	0.1262	0.0261	0.349	1.41E-06
PD-L1	rs822338	9	5451557	t	c	0.1603	0.0276	0.27368	6.46E-09
PD-L1	rs822339	9	5453172	a	g	-0.1716	0.0276	0.26524	5.25E-10
PD-L1	rs860290	9	5453198	a	c	-0.1719	0.0276	0.26493	5.01E-10
PD-L1	rs822340	9	5453260	a	g	-0.1717	0.0278	0.26512	6.31E-10
PD-L1	rs822341	9	5453396	t	c	-0.1731	0.0278	0.2653	4.47E-10
PD-L1	rs822342	9	5453973	t	c	-0.1707	0.0277	0.26512	7.59E-10
PD-L1	rs2282055	9	5455732	t	g	0.1628	0.0278	0.26607	4.90E-09

PD-L1	rs17742278	9	5456523	t	c	0.1583	0.0277	0.27614	1.07E-08
PD-L1	rs1411262	9	5459419	t	c	-0.1657	0.028	0.26669	3.39E-09
PD-L1	rs10114060	9	5461729	a	g	-0.1556	0.0279	0.27124	2.57E-08
PD-L1	rs7041009	9	5463243	a	g	-0.1572	0.028	0.26931	2.09E-08
PD-L1	rs11011802	10	20393945	a	c	-0.3054	0.0665	0.035951	4.37E-06
PD-L1	rs11011804	10	20394779	a	g	0.3091	0.0671	0.0353	4.07E-06

Table S7. *Cis*-pQTLs which related to PD-1/PD-L1 and used in MR analysis

the *reverse* Two-sample-MR with the loose threshold ($P=1\times10^{-5}$,$R^2=0.01$,window=10000)

Exposure	rsid	CHR	POS	effect_allele	other_allele	beta	se	eaf	pval
PD-1	rs10444703	14	38719281	T	C	0.1717	0.0372	0.14077	3.80E-06
PD-1	rs111808226	5	1.35E+08	A	G	0.6272	0.136	0.01036	3.98E-06
PD-1	rs11610127	12	1.15E+08	T	C	0.1369	0.0305	0.22654	7.08E-06
PD-1	rs117444695	6	1.38E+08	A	G	-0.5472	0.1115	0.013056	9.33E-07
PD-1	rs12409262	1	2.46E+08	A	C	0.1733	0.0388	0.155	7.94E-06
PD-1	rs12674255	7	1.32E+08	C	G	0.1143	0.0255	0.44225	7.24E-06
PD-1	rs139693922	8	73944064	T	C	0.2824	0.0633	0.042229	8.13E-06
PD-1	rs144575614	6	1.6E+08	A	G	-0.4079	0.0913	0.026355	7.76E-06
PD-1	rs147652769	2	8057238	T	C	0.5534	0.1068	0.017373	2.19E-07
PD-1	rs189530708	11	1.18E+08	C	G	-0.4134	0.0923	0.021153	7.59E-06
PD-1	rs2330106	22	22716296	C	G	0.3742	0.0399	0.10672	6.17E-21
PD-1	rs34085182	1	2.23E+08	A	G	0.1457	0.0316	0.18442	4.07E-06
PD-1	rs34777990	19	3563017	A	G	0.1367	0.0299	0.26609	4.90E-06
PD-1	rs55720497	16	25686871	A	G	0.4143	0.0832	0.022576	6.31E-07
PD-1	rs71362039	18	8678954	A	C	-0.3356	0.0663	0.038375	4.17E-07
PD-1	rs72692455	4	1.82E+08	A	G	-0.1246	0.027	0.31944	3.98E-06
PD-1	rs7429711	3	24793858	A	C	-0.1107	0.0248	0.48084	7.94E-06
PD-1	rs76144528	3	1.09E+08	A	G	0.3248	0.0716	0.035285	5.75E-06
PD-1	rs77326121	3	73247608	T	C	0.3256	0.0682	0.034474	1.82E-06
PD-1	rs9958590	18	66092216	A	G	-0.1239	0.0271	0.29656	4.90E-06
PD-L1	rs10022491	4	40337908	T	C	-0.1131	0.0254	0.45332	8.32E-06
PD-L1	rs114477766	10	20386048	A	G	-0.3113	0.0676	0.03402	4.17E-06
PD-L1	rs117153321	14	99494928	A	T	0.1709	0.0377	0.13408	5.89E-06
PD-L1	rs12882166	14	1.07E+08	C	G	0.1641	0.0269	0.30042	1.00E-200
PD-L1	rs13322229	3	35068626	T	C	0.1742	0.0369	0.1322	2.40E-06
PD-L1	rs140094912	20	12182666	A	G	-0.3337	0.0719	0.035398	3.55E-06
PD-L1	rs141530216	11	25295091	A	T	0.3934	0.0872	0.02316	6.46E-06
PD-L1	rs188070839	5	31955596	A	G	0.3872	0.086	0.024303	6.76E-06
PD-L1	rs188907004	19	5570275	T	C	-0.6806	0.1524	0.007271	7.94E-06
PD-L1	rs2634828	2	36263686	A	T	-0.1168	0.0258	0.35213	6.03E-06
PD-L1	rs28363920	4	20074331	T	C	0.3626	0.081	0.024796	7.41E-06
PD-L1	rs28836477	16	58389301	A	G	-0.1223	0.0275	0.33361	8.71E-06
PD-L1	rs34803358	8	13533716	A	G	0.3799	0.0846	0.024712	7.08E-06
PD-L1	rs6914467	6	12864309	A	G	-0.232	0.0517	0.065753	7.08E-06
PD-L1	rs75073941	3	72671327	T	C	0.4188	0.0947	0.020236	9.77E-06
PD-L1	rs75176410	10	84921747	T	C	0.4097	0.0902	0.022463	5.50E-06
PD-L1	rs77519204	18	41586957	T	G	-0.314	0.0688	0.033323	5.13E-06
PD-L1	rs79643961	10	428383	A	G	-0.2132	0.0479	0.071531	8.32E-06
PD-L1	rs79906842	2	1.74E+08	T	G	0.2815	0.0623	0.042752	6.31E-06
PD-L1	rs9859911	3	1.08E+08	A	G	-0.1981	0.0417	0.098796	2.09E-06

the *reverse* Two-sample-MR with the loose threshold ($P=5\times10^{-6}$,$R^2=0.001$,window=10000)

Exposure	rsid	chromosome	position	effect_allele	other_allele	beta	se	eaf	pval
PD-1	rs34085182	1	2.23E+08	A	G	0.1457	0.0316	0.18442	4.07E-06
PD-1	rs147652769	2	8057238	T	C	0.5534	0.1068	0.017373	2.19E-07
PD-1	rs77326121	3	73247608	T	C	0.3256	0.0682	0.034474	1.82E-06
PD-1	rs72692455	4	1.82E+08	A	G	-0.1246	0.027	0.31944	3.98E-06
PD-1	rs111808226	5	1.35E+08	A	G	0.6272	0.136	0.01036	3.98E-06
PD-1	rs117444695	6	1.38E+08	A	G	-0.5472	0.1115	0.013056	9.33E-07
PD-1	rs10444703	14	38719281	T	C	0.1717	0.0372	0.14077	3.80E-06
PD-1	rs55720497	16	25686871	A	G	0.4143	0.0832	0.022576	6.31E-07
PD-1	rs71362039	18	8678954	A	C	-0.3356	0.0663	0.038375	4.17E-07
PD-1	rs9958590	18	66092216	A	G	-0.1239	0.0271	0.29656	4.90E-06
PD-1	rs34777990	19	3563017	A	G	0.1367	0.0299	0.26609	4.90E-06
PD-1	rs2330106	22	22716296	C	G	0.3742	0.0399	0.10672	6.17E-21
PD-L1	rs114477766	10	20386048	A	G	-0.3113	0.0676	0.03402	4.17E-06
PD-L1	rs12882166	14	1.07E+08	C	G	0.1641	0.0269	0.30042	1.00E-200
PD-L1	rs13322229	3	35068626	T	C	0.1742	0.0369	0.1322	2.40E-06
PD-L1	rs140094912	20	12182666	A	G	-0.3337	0.0719	0.035398	3.55E-06
PD-L1	rs9859911	3	1.08E+08	A	G	-0.1981	0.0417	0.098796	2.09E-06

Table S8. The Species-level MR result with the loose threshold ($P=1\times10^{-5}$,$R^2=0.01$,window=10000)

	Outcome	Exposure	variants		R2	F	random IVW			Weighted median		MR Egger			Weighted Mode		Q		MR presso
Forward			nSNP	outlier			beta 95% CI	P	P BH	Beta	P	Beta	P	Beta	P intercept	P	Beta	Q	IVW-Q_	Gable-p
	PD-L1	s_Coprococcus_catus	3	0	0.02	21-21.3	0.1 (-0.2 to 0.4)	0.600	0.600	0.078	0.450	0.140	0.517	-0.677	0.475	0.441	0.229	1.970	0.373	-
		s_Coprococcus_comes	8	0	0.04	21.4-26.7	-0.1 (-0.3 to 0.1)	0.234	0.586	-0.111	0.448	-0.088	0.789	0.155	0.644	0.756	-0.054	2.184	0.949	0.951
		s_Coprococcus_sp_ART55_1	3	0	0.07	21.1-22.4	0 (-0.2 to 0.2)	0.651	0.743	-0.031	0.693	0.038	0.355	0.490	0.330	0.618	0.062	3.805	0.149	
		s_Ruminococcus_bromii	4	0	0.02	21.3-24.7	-0.1 CI (-0.4 to 0.1)	0.405	0.910	-0.106	0.873	-0.024	0.867	-0.129	0.975	0.910	0.026	2.182	0.536	0.570
		s_Ruminococcus_callidus	3	0	0.03	21.4-23.5	-0.1 (-0.3 to 0.2)	0.627	0.937	-0.056	0.937	0.012	0.377	-1.074	0.390	0.795	0.052	2.038	0.361	-

		s_Ruminococcus_lactaris	2	0	0.03	21.5-23.2	0.2 (-0.1 to 0.6)	0.147	0.147	0.249	-	-	-	-	-	-	-	0.507	0.476	-
		s_Ruminococcus_obeum	8	2	0.04	21.1-30	0 (-0.2 to 0.2)	0.749	0.749	-0.031	0.539	-0.084	0.529	-0.267	0.564	0.506	-0.145	6.938	0.435	0.442
		s_Ruminococcus_torques	5	0	0.03	21.1-26.1	0.1 (-0.1 to 0.3)	0.592	0.592	0.056	0.375	0.122	0.429	-0.493	0.376	0.544	0.129	3.318	0.506	0.529
	**PD-1**	s_Prevotella_copri	4	0	0.02	21.4-22.5	-0.1 (-0.4 to 0.2)	0.463	0.505	-0.105	0.404	-0.139	0.388	-0.740	0.438	0.505	-0.192	3.222	0.359	0.410
**Reverse**	**PD-L1**	s_Odoribacter_splanchnicus	7	0	0.14	19.7-22.6	0 (-0.1 to 0.1)	0.764	0.873	-0.019	0.873	-0.013	0.650	-0.111	0.694	0.842	0.022	2.086	0.861	0.913
		s_Parabacteroides_distasonis	6	1	0.14	19.8-22.6	0.1 (0 to 0.2)	0.156	0.391	0.092	0.402	0.069	0.801	0.068	0.927	0.730	0.045	3.561	0.614	0.631
		s_Parabacteroides_goldsteinii	7	0	0.14	19.5-37.2	-0.1 (-0.4 to 0.1)	0.293	0.621	-0.124	0.713	-0.057	0.373	-0.431	0.502	0.837	-0.047	3.193	0.784	0.783
		s_Parabacteroides_johnsonii	7	0	0.14	19.8-22.6	0 (-0.2 to 0.3)	0.900	0.900	0.015	0.729	0.056	0.614	-0.247	0.579	0.759	0.068	1.530	0.957	0.956
		s_Parabacteroides_merdae	6	1	0.14	19.8-22.6	0.1 (-0.1 to 0.2)	0.272	0.378	0.072	0.207	0.104	0.710	0.108	0.898	0.303	0.137	4.433	0.489	0.519
		s_Parabacteroides_unclassified	6	1	0.14	19.8-22.6	0.2 (0 - 0.4)	0.012	0.029	0.209	0.801	0.152	0.748	0.561	0.311	0.436	0.132	2.290	0.808	0.827
