# Supplementary figures and images for: Causal relationships between gut microbiota and programmed cell death protein 1/programmed cell death-ligand 1: A bidirectional Mendelian randomization study
Source: Front Immunol. 2023 Mar 9;14:1136169. doi: 10.3389/fimmu.2023.1136169 (PMC10034163; doi:10.3389/fimmu.2023.1136169)

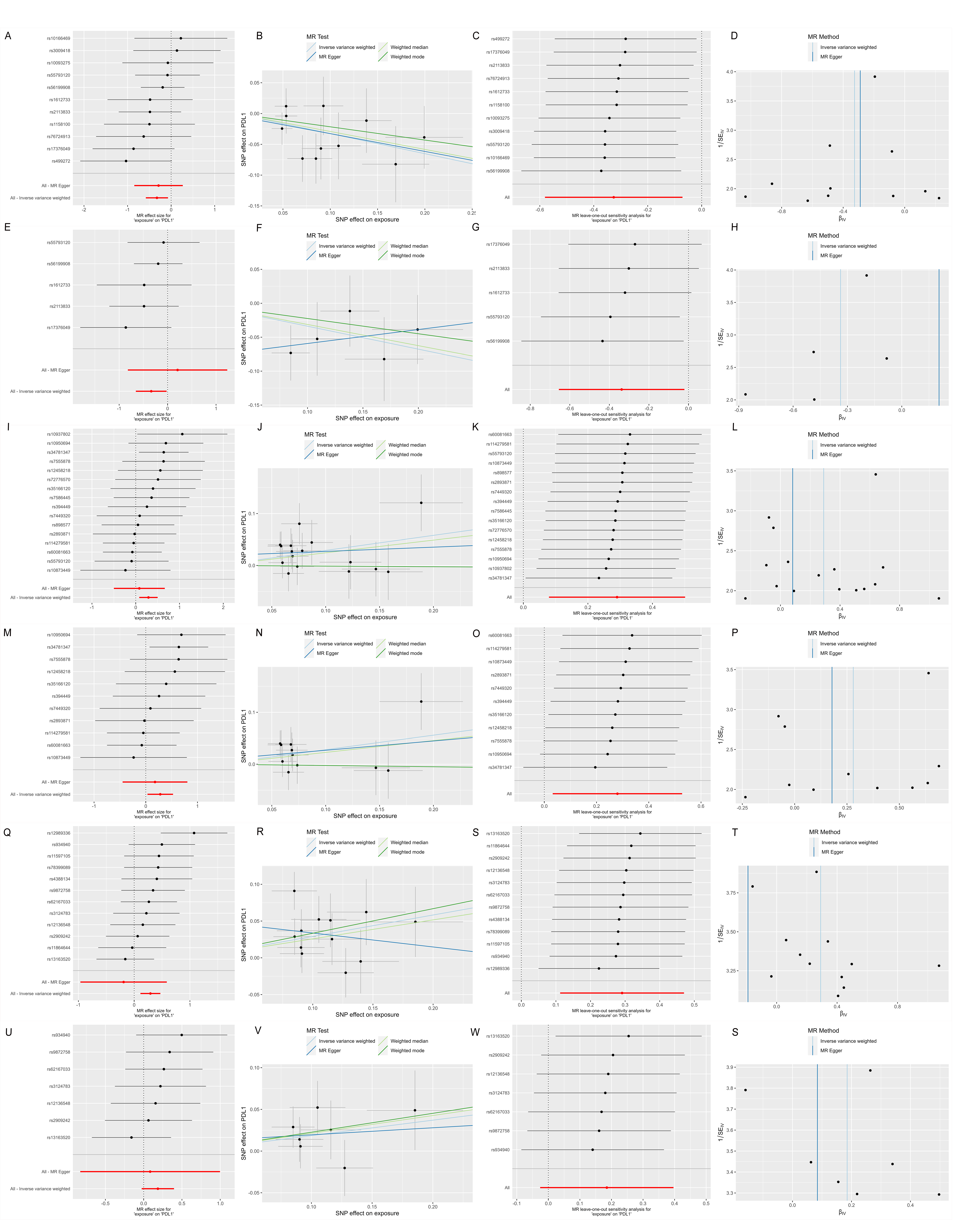

Supplement: Supplementary file 2 [file Image_1.tif]

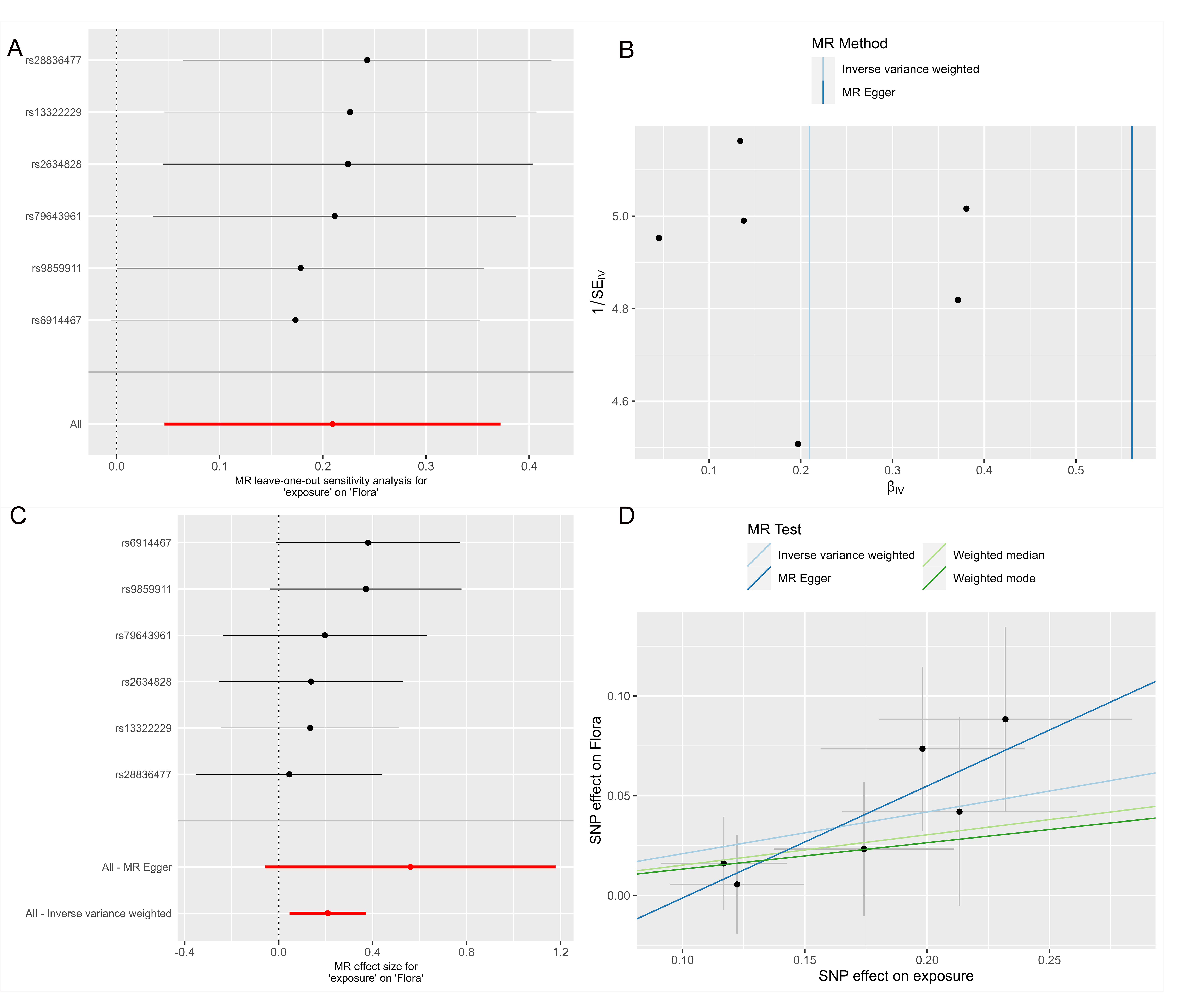

Supplement: Supplementary file 3 [file Image_2.tif]

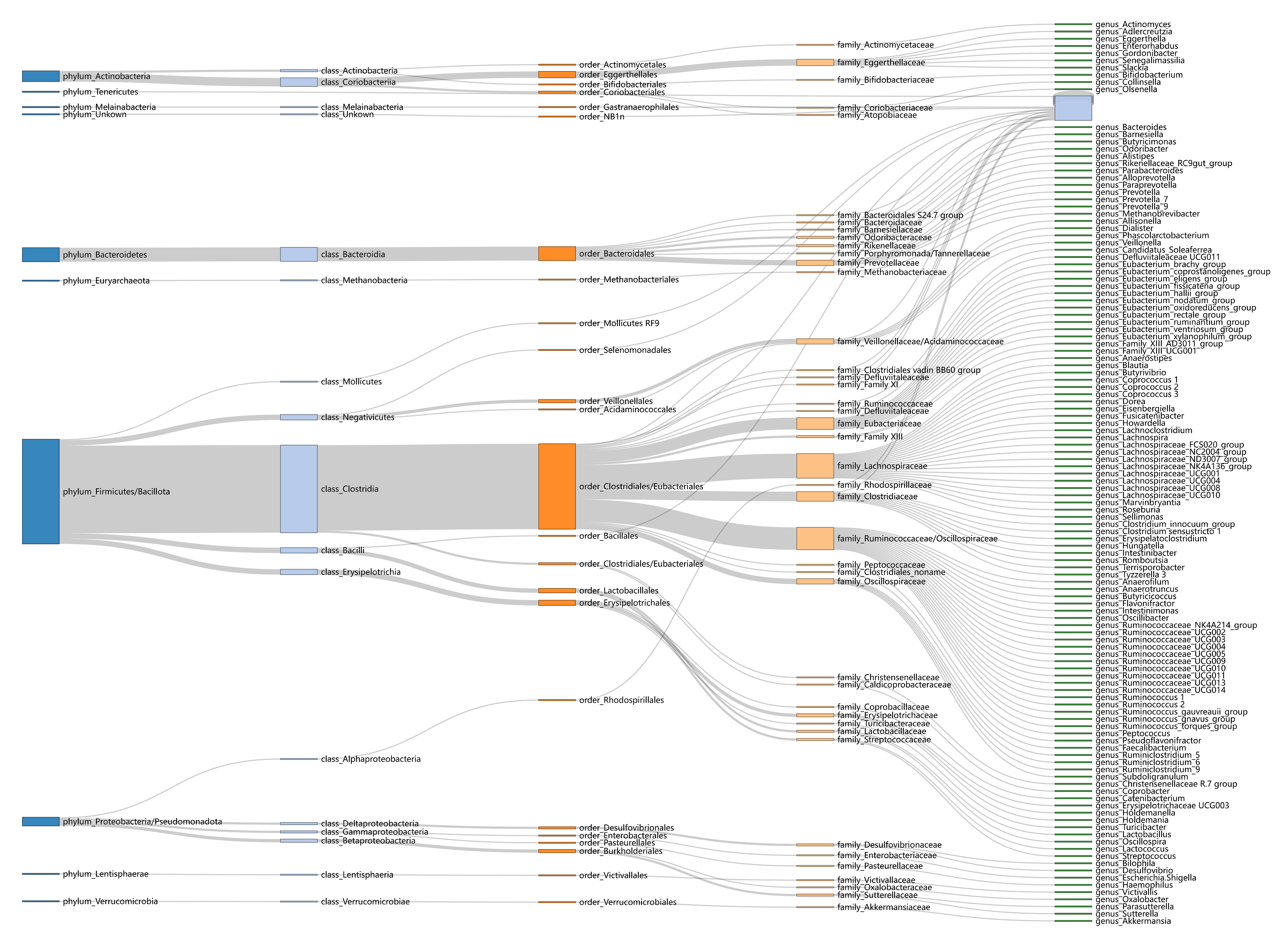

Supplement: Supplementary file 4 [file Image_3.tif]

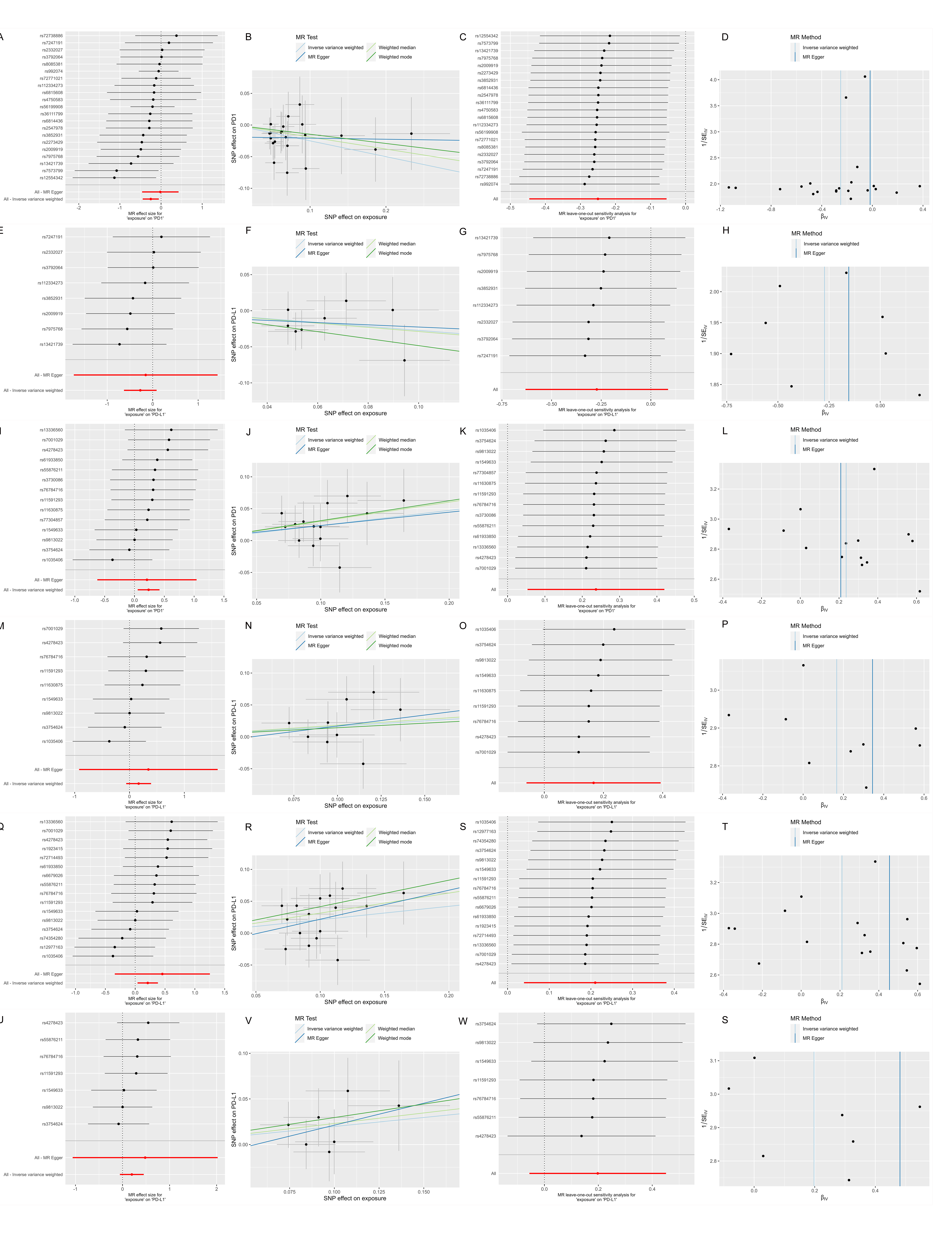

Supplement: Supplementary file 5 [file Image_4.tif]

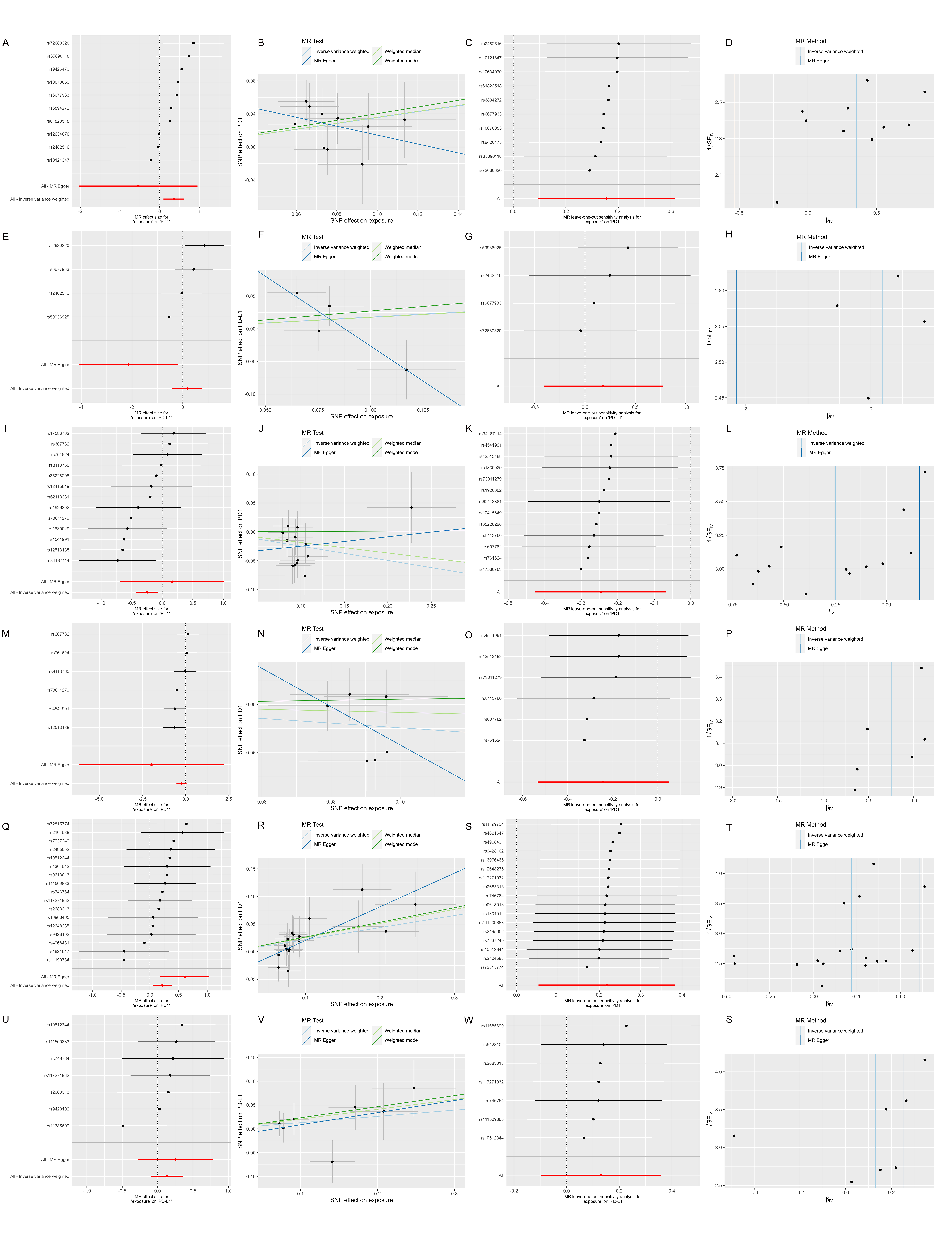

Supplement: Supplementary file 6 [file Image_5.tif]
